# Ubiquitousness of *Haloferax* and Carotenoid Producing Genes in Arabian Sea Coastal Biosystems of India

**DOI:** 10.3390/md19080442

**Published:** 2021-07-31

**Authors:** Jamseel Moopantakath, Madangchanok Imchen, Ranjith Kumavath, Rosa María Martínez-Espinosa

**Affiliations:** 1 Department of Genomic Science, School of Biological Sciences, Central University of Kerala, Tejaswini Hills, Periye 671320, Kerala, India; mailforjamseel@gmail.com (J.M.); anokimchen@gmail.com (M.I.); 2 Biochemistry and Molecular Biology Division, Agrochemistry and Biochemistry Department, Faculty of Sciences, University of Alicante, Ap. 99, E-03080 Alicante, Spain; rosa.martinez@ua.es; 3 Multidisciplinary Institute for Environmental Studies “Ramón Margalef”, University of Alicante, Ap. 99, E-03080 Alicante, Spain

**Keywords:** halophiles, haloarchaea, carotenoid, microbial pigments, *Haloferax*

## Abstract

This study presents a comparative analysis of halophiles from the global open sea and coastal biosystems through shotgun metagenomes (*n* = 209) retrieved from public repositories. The open sea was significantly enriched with *Prochlorococcus* and *Candidatus pelagibacter*. Meanwhile, coastal biosystems were dominated by *Marinobacter* and *Alcanivorax*. Halophilic archaea *Haloarcula* and *Haloquandratum*, predominant in the coastal biosystem, were significantly (*p* < 0.05) enriched in coastal biosystems compared to the open sea. Analysis of whole genomes (*n* = 23,540), retrieved from EzBioCloud, detected *crtI* in 64.66% of genomes, while *cruF* was observed in 1.69% Bacteria and 40.75% Archaea. We further confirmed the viability and carotenoid pigment production by pure culture isolation (*n* = 1351) of extreme halophiles from sediments (*n* = 410 × 3) sampling at the Arabian coastline of India. All red-pigmented isolates were represented exclusively by *Haloferax*, resistant to saturated NaCl (6 M), and had >60% G + C content. Multidrug resistance to tetracycline, gentamicin, ampicillin, and chloramphenicol were also observed. Our study showed that coastal biosystems could be more suited for bioprospection of halophiles rather than the open sea.

## 1. Introduction

Carotenoids are natural pigments produced by plants, microbes, some fungi and microalgae. More than 750 carotenoids of potential commercial importance have been isolated from microbial sources [1]. In particular, Haloarchaea is a reservoir of unique carotenoid bacterioruberin [2]. Carotenoids synthesized by extreme halophiles are of specific interest for their ease in the extraction process, for saline tolerance, and for their biological applications against infectious diseases, repression of tumors or cancer growth, and cancer growth [3,4,5,6]. These extreme halophiles are mainly predominant in marine biosystems such as the open sea and coastal regions (salty marshes, salted ponds or similar ecosystems) [7].

Marine biosystems are exposed to unique environmental stress based on geographical location. For instance, coastal biosystems are exposed to continuous abiotic fluctuations such as salinity, pH, temperature, nutrients, and light, which enrich stress-tolerant enzymatic systems [8,9,10,11]. In contrast to coastal biosystems with biogenic and abiogenic colonizable particles, the open sea is rich in biogenic particles [12]. For instance, the open ocean has a lower abundance of available iron, which has led to the significant reduction of iron stress genes in *Synechococcus* strains from the open sea compared to coastal regions [13]. The open sea is also a rich reservoir of bacteria representing over 90% of total marine biomass [14]. The open sea is significantly influenced by seasonal and temperature changes [15], and microbial communities that are the primary energy producers [16].

On the other hand, coastal biosystems are interconnected with freshwater bodies including rivers that constantly carry foreign solutes and microbes into the marine biosystem that could enrich the diverse microbial community. In our recent study, we detected the predominance of Haloarchaea in the Arabian Sea coastal region, India [2]. Compared to nearshore water, the open sea is also exposed to higher Ultraviolet (UV) radiation due to more increased transparency because of lower amounts of suspended particles [17]. Nevertheless, the sediment in the shallow coastal environment is exposed to similar UV radiation and higher salinity. Carotenoid pigments such as bacterioruberin are known to provide metabolic balance via osmoregulation and oxidation stress, respectively, when exposed to salinity and UV radiation [18,19,20,21]. Despite the differences between the open sea and coastal region, there is no in-depth comparative study on the diversity of pigmented halophiles to the best of our knowledge. Hence, this study attempts to perform comprehensive research to delineate the microbiome and halophilic microbial community differences between the open and coastal regions. Subsequently, we isolated halophiles and profiled their potential phenotypical, biochemical, and carotenoid production based on the findings.

## 2. Results

### 2.1. Microbiome Composition in Open Sea and Seacoast

**Figure 1:**
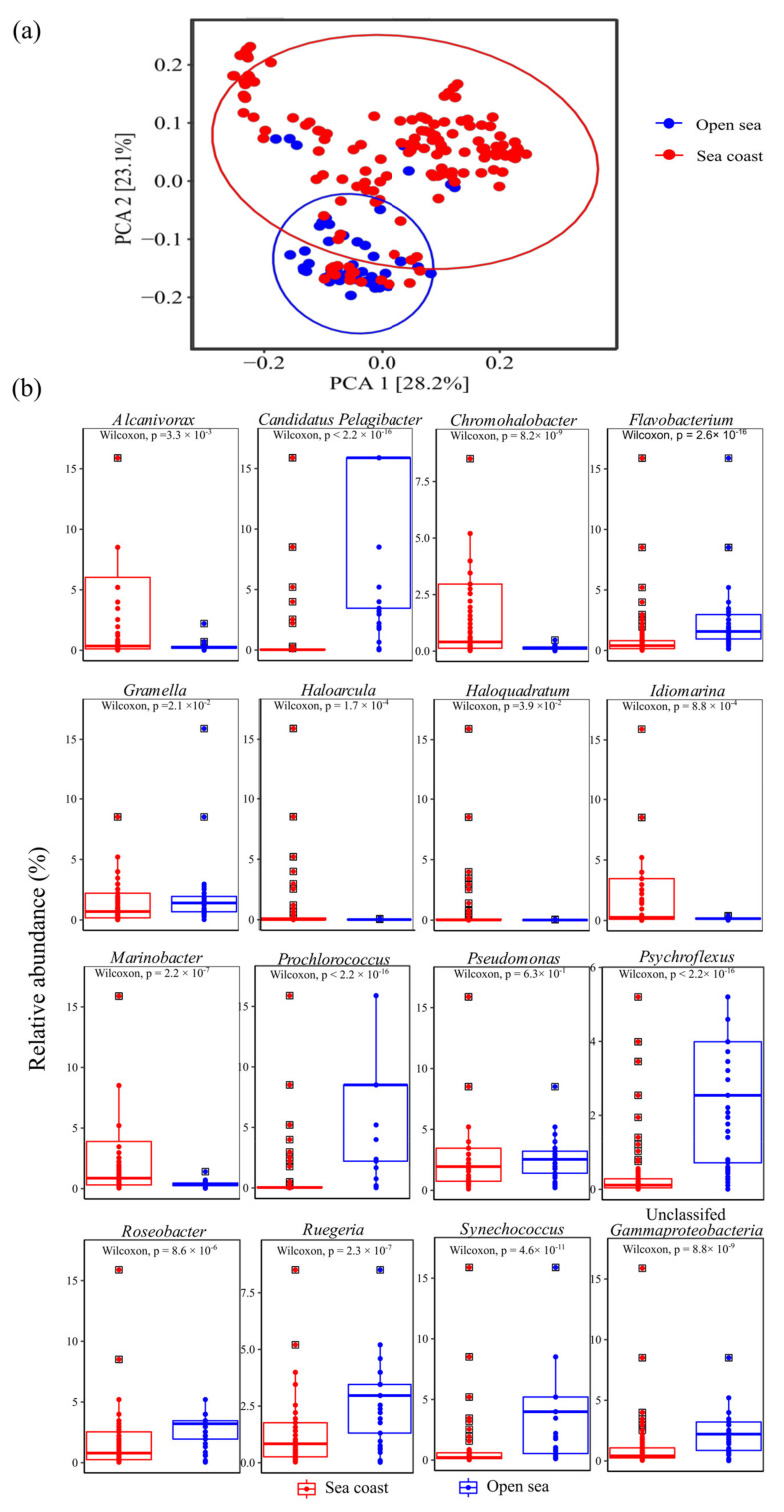
(**a**) Weighted UniFrac PCA plot of the open sea and coastal microbiome. (**b**) Cumulative predominant microbial genera were plotted by retrieving the top 10 genera in the open sea and coastal biosystems.

The open sea and coastal biosystems were composed of 665 operational taxonomic units (OTUs). The microbiome composition between the biosystems was significantly different, which is evident from the separate clustering of the biosystems in the Weighted UniFrac Principal component analysis (PCA) (Figure 1a). Interestingly, several metagenomes from both biosystems overlapped, suggesting similarities between the datasets. To identify the genera contributing to the similarities and differences, we analyzed the top 10 dominant genera in both ecosystems (Figure 1b). *Flavobacterium* and *Gramella* genera were common to both biosystems with strong similarities regarding their abundance. However, the open sea was significantly enriched with *Prochlorococcus* and *Candidatus Pelagibacter*.

In contrast, the coastal biosystems were dominated by *Pseudomonas*, *Marinobacter* and *Alcanivorax*. In addition, the halophilic archaea *Haloarcula* and *Haloquadratum* were also represented among the top 10 predominant genera in the coastal biosystem with significant (*p* < 0.05) enrichment compared to the open sea (Figure 1b). Surprisingly, in the open sea, extreme halophiles were not detected among the top 10 genera. The predominance of the coastal biosystems by halophiles, particularly those belonging to the haloarchaeal genera, could suggest that coastal biosystems are a better avenue for bioprospection of halophiles.

### 2.2. Diversity of Halophilic Microbiome in Open Sea and Seacoast

**Figure 2:**
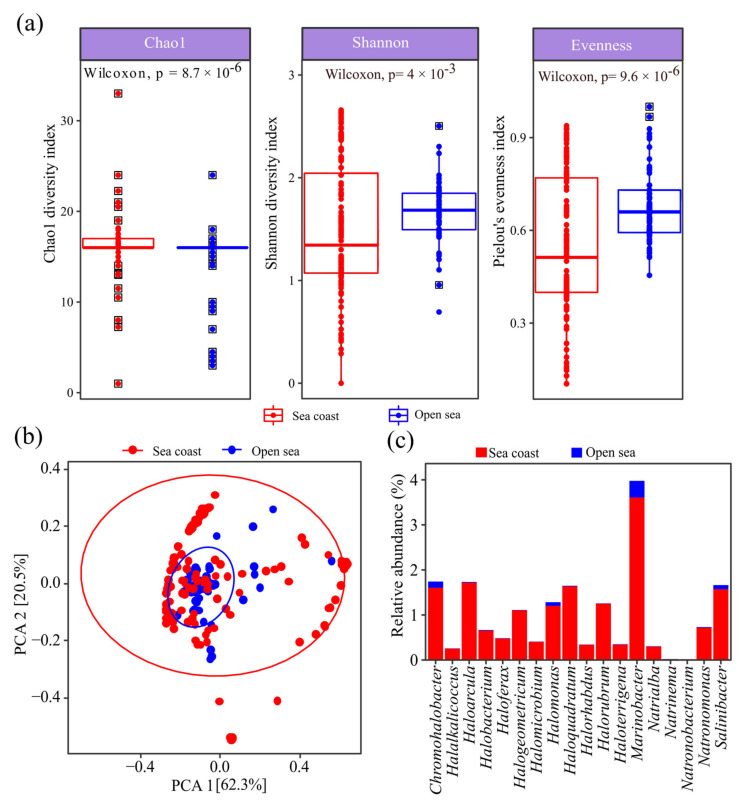
(**a**) Alpha diversity, (**b**) Weighted UniFrac PCA plot and (**c**) relative abundance of the extreme halophilic microbial community in the open sea and coastal metagenomes.

The diversity of the halophiles was further investigated by determining the alpha diversity and composition of the halophiles. Chao1 diversity estimation indicated moderately higher diversity (*p* < 0.05) in the coastal ecosystem compared to the open sea (Figure 2a).

On the contrary, the Shannon index and Pielou’s evenness were moderately higher in the open sea, suggesting that the abundance of halophiles in the coastal ecosystem could be unevenly distributed. The weak differences in diversity between the biosystems were further observed in the Weighted UniFrac PCA analysis (Figure 2b). Interestingly, the similarities observed between the microbiome were due to the similarities in the proportion of predominant OTUs. This indicates that halophiles between the biosystems share similarities in diversity yet differences in relative abundance (Figure 2c).

### 2.3. Diversity of Carotenoid Gene in the Halophiles

**Figure 3:**
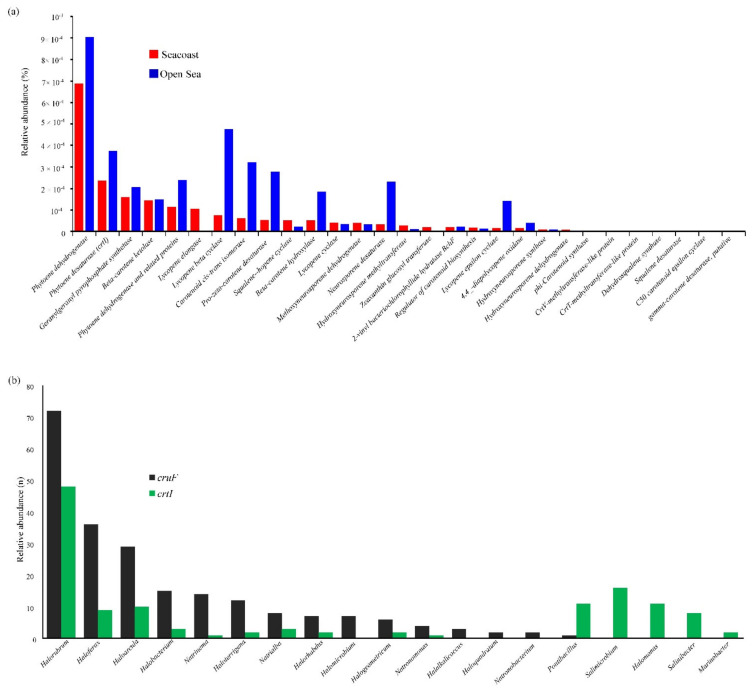
(**a**) Relative abundance (%) of a functional gene within carotenoid metabolism in subsystems level 3 classification. (**b**) *crtI* and *cruF* gene abundance of the whole genome of extreme halophiles detected within the pan-metagenome.

Functional genes (*n* = 31) in the carotenoid synthesis pathways were similarly distributed in the open sea and seacoast (Figure 3a).

Genes encoding phytoene dehydrogenase, responsible for the lycopene synthesis, were predominant in both biosystems. Despite the significant difference in the functional potential for carotenogenesis, the whole metagenome dataset could provide a biased snapshot for functional genes with low abundance due to insufficient sequencing depth.

Hence, the whole genome of 23,540 species was retrieved from a public repository to circumvent these weaknesses, and the prevalence of significant carotenoid genes viz. *crtI*, *crtL*, *cruF*, and *crtD* genes, were investigated. A total of 64.66% of species harbored the *crtI* carotenoid gene, indicating wide prevalence (Table 1). The *crtI* (phytoene desaturase) and *cruF* (C_50_ carotenoid 2″,3″-hydratase) genes were predominant in *Halorubrum* and *Haloferax* genera. The abundance and distribution of *crtI* and *cruF* gene significantly differed between genera (Figure 3b).

### 2.4. Isolation of Pigmented Halophiles across the Arabian Sea Coastline

**Figure 4:**
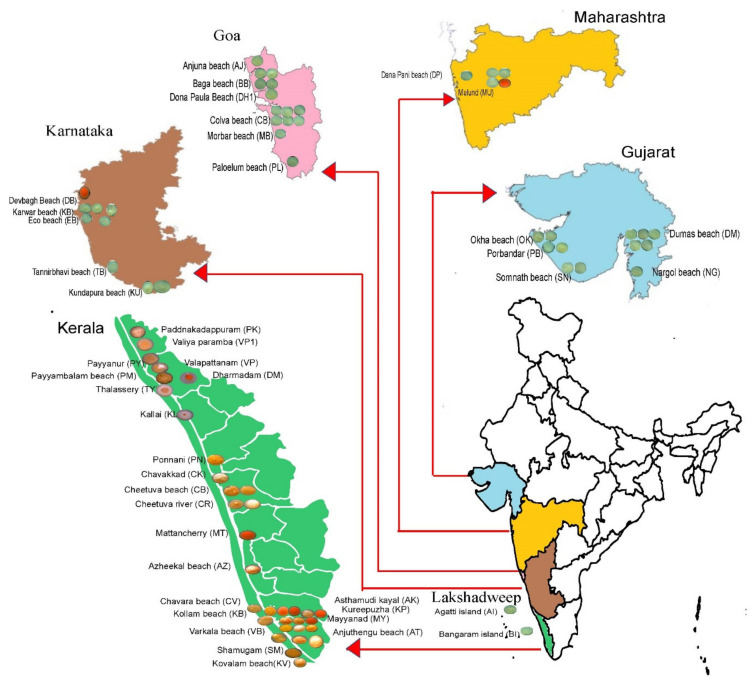
Graphical representation of sediment sampling locations along the Arabian coastline. The stereomicroscopic image of the extreme halophilic pure cultures are shown within the inset map.

As per the metagenomic analysis, coastal biosystems represent a more suitable avenue for the isolation of halophiles than the open sea. In addition, whole-genome analysis further indicated the prevalence of carotenoid genes among the halophiles, indicating the prevalence of carotenoid synthesis. These observations warrant further confirmation through a culture-dependent approach to determine the cultivability and carotenoid production. Since the metagenome was retrieved from public datasets, obtaining the sediments was out of the scope of this study. Hence, coastal sediments were collected in triplicates (*n* = 410 × 3) along the Arabian coastline of India from seashore (*n* = 202), estuaries (*n* = 25), rivers (*n* = 86), mangroves (*n* = 51), lakes (*n* = 28), island (*n* = 15), and saltpan (*n* = 3). Halophilic pure cultures (*n* = 1351) were obtained by enriching the sediments in MSG (Modified Sehgal and Gibbon’s) medium with saturated 6M NaCl. Red, yellow, or orange pigmentation was exhibited by 77 isolates (5.69%) (Figure 4). The yellow-pigmented isolates were the most abundant (66.23%), followed by orange (20.78%) and red (12.99%) pigments (Figure S1a). Among the yellow-pigmented isolates, 78.43% originated from seashore sediments, and 1–5% were from other biosystems such as a river, saltpan, island, mangrove, and lakes. Red-pigmented halophiles were isolated from all the biosystems except island samples. The red-pigmented isolates were especially obtained from mangrove sediments (30%). River and lake sediments constituted 20% of the overall red-pigmented isolates, while saltpan and estuary harbored only 10% of the red-pigmented isolates. Interestingly, orange pigmented halotolerant were only observed in the three biosystems, i.e., seashore, river, and mangrove (56.25%, 25%, and 18.75% respectively) (Figure S1b). Sediments from Kerala had the highest evenness in the distribution of pigmented isolates, i.e., yellow (29.41%), orange (47.05%), and red (23.52%). In contrast, sediments from Karnataka, Maharashtra, Goa, and Gujarat were dominated (80–100%) with light, yellow-pigmented isolates. The red pigmented isolates could be isolated mainly from Kerala (*n* = 8), followed by Maharashtra (*n* = 1) and Karnataka (*n* = 1) (Figure S1c).

### 2.5. Morphological Diversity across the Halotolerant Organism

Gram-positive isolates were dominant (66.23%) among the isolates. Rod-shaped Gram-positive, rod-shaped Gram-negative, and Gram-positive cocci accounted for 32.46%, 31.16%, and 32.46%, respectively. The rare ones were Gram-positive coccobacillus (1.29%) and Gram-negative irregular cocci (1.29%). Furthermore, it was observed that 77.92% of the isolates were motile, and 46.75% of isolates could survive in both aerobic and aerobic conditions (Table S1).

### 2.6. Biochemical Diversity across the Halotolerant Organisms

Urease activity was detected in 66.23% of the isolates. Glucose, as the primary source of carbon, was observed in 46.75% of the isolates. Mannitol, lactose, and sorbitol utilization was observed in 36.36%, 32.46%, and 28.57% of the isolates, respectively. Furthermore, oxidase (45.45%) and catalase activities (35.06%) were also widespread among the halophiles. A significant (*p*_Kruskal–Wallis_ =1.9 × 10^−9^) difference was observed in the biochemical properties of the isolates based on locations. Out of 32 biochemical tests, isolates RK_AK2, RK_AZ, RK_CR2, RK_KB, and RK_MT from Kerala were positive for 46.87%, 37.5%, 34.37%, 34.37%, and 31.25% of the biochemical test, respectively (Table S2). Furthermore, isolates from Kerala exhibited a similar pattern of oxidase (61.76%) and urease activity (61.76%). All isolates from Lakshadweep showed urease, glucose, catalase, lactose and sorbitol activities. H_2_S-producing isolates were observed from Karnataka (44.44%), Gujarat (23.07%), and Goa (21.42%). Furthermore, 14–23% esculin activity was also found in Kerala, Karnataka, Gujarat, and Goa isolates.

### 2.7. Antibiotic Susceptibility Profiling

Most isolates (61.03%) were resistant to gentamicin at a concentration of 25 µg/mL, and 36.36% were resistant to the antibiotics at concentrations up to 100 µg/mL. Similarly, 46.75% of isolates were resistant to tetracycline at concentrations up to 25 µg/mL. However, the isolates were susceptible to ampicillin (72.73%) and chloramphenicol (76.63%) at a concentration of 100 µg/mL (Figure S1d).

### 2.8. Influence of NaCl Concentration on Pigment Production

The microbial isolates were resistant to 3–6 M NaCl, thus showing an extreme halophilic profile. Interestingly, 94% of the 6 M NaCl resistant halophiles were isolated from Kerala, and 3% of isolates were from Maharashtra and Karnataka. Isolates from Goa and Gujarat were resistant to a maximum salinity of 3 M NaCl (a,b). Furthermore, all isolates with red and orange pigments were resistant to 6M NaCl, whilst only 20% of the yellow-pigmented isolates were resistant to 6 M NaCl (Figure S2c). Pigment production was observed after 50 h. incubation and attained optimum levels after 300 h. incubation (Figure S2f,g).

### 2.9. Identification of the Isolates through 16S rDNA Phylogenetic Analysis

Phylogenetic analysis revealed that 23 isolates belong to Haloarchaea and 54 isolates belong to the bacterial domain (Table S3). In contrast to Haloarcheal diversity observed through the metagenomic approach, the Haloarchea obtained through culturable technique was represented solely by *Haloferax* species such as *Haloferax alexandrinus, Haloferax lucentense, Haloferax chudinovii,* and *Haloferax sulfurifontis.* Halophilic bacterial isolates were represented by *Chromohalobacter, Salimicrobium, Halomonas, Pontibacillus, Staphylococcus, Virgibacillus, Lysinibacillus, Pseudomonas, Bacillus,* and *Acidovorax* genera (Figure S3a–c). Interestingly, 91.3% of the total haloarchaea were isolated from Kerala, whereas only 4.3% were isolated from Maharashtra and Karnataka. Halophilic bacteria were most isolated from Goa (25.92%), Kerala (24.07%), and Gujarat (24.07%). Lakshadweep samples had the lowest percentage of halophilic archaea or bacteria (3.70%). A significant difference in genera distribution was observed between the locations (*p*_Kruskal–Wallis_ = 0.02).

**Figure 5:**
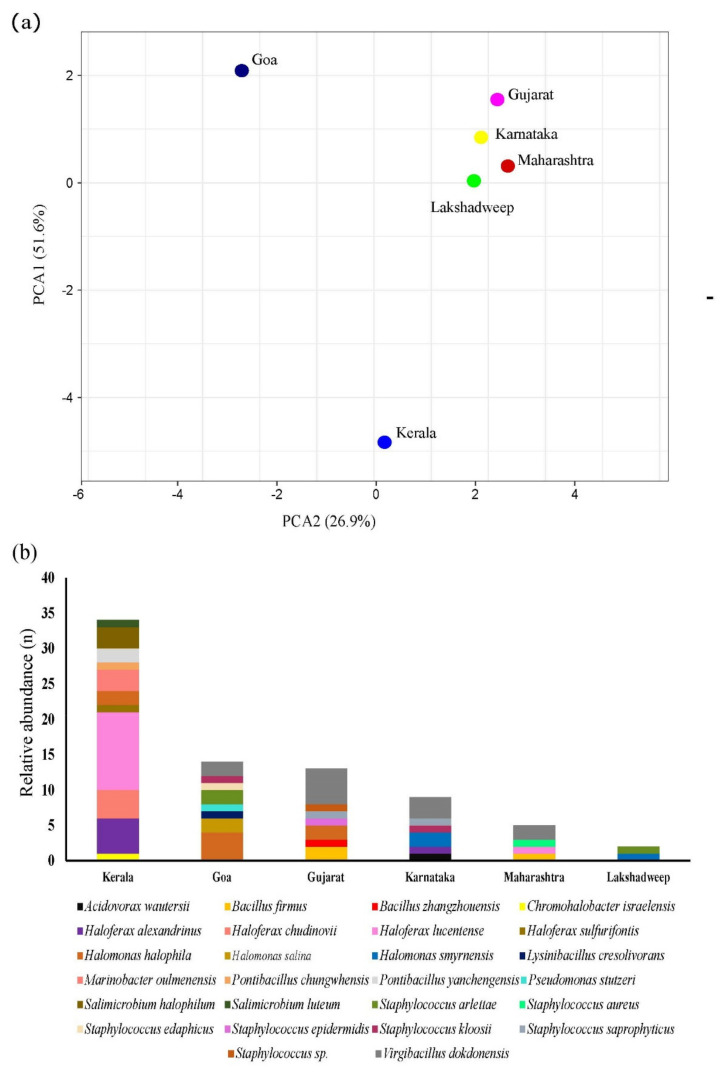
(**a**) PCA plot and (**b**) stacked bar chart of the culturable extreme halophiles isolated from different locations.

Isolates from Gujarat samples were represented by *Virgibacillus* (38.46%), *Staphylococcus* (23.07%), *Bacillus* (23.07%), and *Halomonas* (15.38%). Isolates from Goa were represented by well-known salt-tolerant *Halomonas* (42.85%), followed by *Virgibacillus* (14.28%) and *Staphylococcus* (28.57%). Interestingly, *Lysinibacillus* (7.14%) and *Pseudomonas* (7.14%) were isolated only from the Goa. Sediment from Maharashtra was predominated with *Virgibacillus* (40%), followed by *Haloferax, Staphylococcus,* and *Bacillus*. Lakshadweep sediments were enriched with only two halotolerant organisms, i.e., *Halomonas* and *Staphylococcus*. Kerala biosystem had the highest haloarchaea abundance (61.76% *Haloferax*) in addition to diverse bacterial genera such as *Chromohalobacter, Marinobacter, Salimicrobium,* and *Pontibacillus*. A significant difference in species abundance was observed between geographical locations based on Kruskal–Wallis test (*p* < 0.03) and principal component analysis (Figure 5). *H. lucentense*, *H. alexandrinus,* and *H. chudinovii* were enriched in Kerala, while *Halomonas halophila* was enriched in Gujarat, Goa, and Kerala. (Figure 5). Comparative analysis showed a significant difference in species distribution in each biosystem (Kruskal–Wallis *p* = 1.31 × 10^−17^).

**Figure 6:**
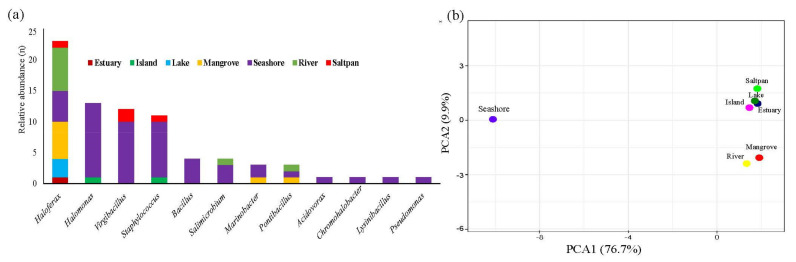
Genera-level distribution of the culturable extreme halophiles represented in (**a**) stacked bar chart and (**b**) PCA plot based on biosystems.

Sediments from seashore were represented in a distinct axis in the PCA analysis, enriched with twenty-four diverse species with dominant *Virgibacillus dokdonensis*. However, estuary, island, lake, and saltpan clustered together (Figure 6a,b). Within the *Haloferax* community, *H. lucentense* was predominant in mangroves and rivers.

### 2.10. G + C Diversity across the Halotolerant Organisms

A significant difference in the G + C content was observed between Haloarchaeal and Halobacterial isolates (Epps-singleton *p* = 6.3427 × 10^−12^) in line with the results reported in the literature [18,19]. The G + C content for 86.95% of the Haloarcheal isolates was above 60%, whereas only 36.36% of Halobacteria had G+C contents above 60% (Figure S2d,e).

## 3. Discussion

Marine ecosystems represent one of the largest biosystems with an enormous untapped resource of bioactive molecules. Carotenoids of marine origin have gained interest for their multitude of applications in industrial and pharmaceutical areas. The diversity of carotenoid synthesis genes in marine microbes can vary substantially between biosystems. However, to the best of our knowledge, a broad-scale analysis of the carotenoid synthesis gene diversity between the open sea and coastal regions has never been carried out. In this study, we performed a comparative shotgun metagenome meta-analysis of open sea and coastline halophilic community and further confirmed it through pure culture isolation.

**Figure 7:**
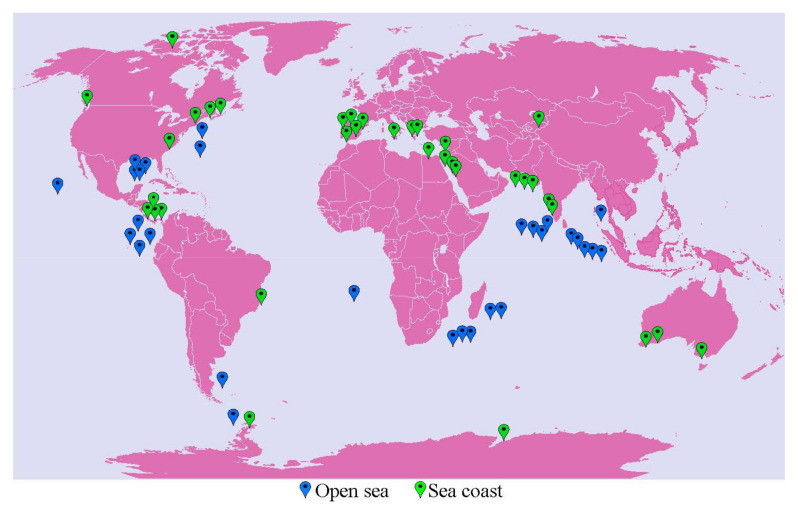
Global map representing the sampling location of the metagenomes retrieved from the MG-RAST server. Sampling sites of coastal and open sea metagenome datasets are highlighted in green and blue, respectively (the global map was generated using https://maps.com, accessed on 7 March 2021).

We analyzed the global marine biosystems through shotgun metagenome data (*n* = 209) and whole-genome (*n* = 23,540) (see Figure 7 in Materials and Methods section and Table S4). Furthermore, we implemented the culturable technique to isolate halophiles from the Arabian Sea coast of India. As per the metagenomic analysis, halophiles were most diverse in the seacoast biosystems. The carotenoid synthesis genes *crtI* and *cruF* were predominant in the *Halorubrum* and *Haloferax* genera. The significant enrichment of halophiles in coastal biosystems could be due to the salt accumulation upon marine water evaporation [10]. Similarly, the predominance of *Haloferax* was also observed based on culturable techniques. The halophilic isolates from coastal sediments (*n* = 410 × 3) were further investigated to evaluate the culturable diversity of extreme halophiles (carotenoid production, phenotypic, biochemical, and genotypic characteristics).

### 3.1. Extreme Halophiles Are Enriched in Seacoast Biosystems

The microbiome structure differed significantly between the open sea and coastal metagenome datasets, although some similarities were also observed. The open sea surface is constantly exposed to sunlight, which provides a suitable condition for photosynthetic bacteria. This leads to the enrichment of photosynthetic genera *Prochlorococcus* (7.8 ± 0.75%) and *Synechococcus* (4.03 ± 0.52%) in the open sea [22]. Furthermore, the unrestricted availability of organic matter enriches *Pelagibacter* (10.5 ± 0.86%), which feeds on organic matter in the open sea. In contrast, the coastal biosystems were enriched with *Alcanivorax* and *Marinobacter,* which are well-known hydrocarbon-degradation indicators [23,24]. In addition, phosphate solubilizer and nitrogen-fixing genera *Pseudomonas* were highly enriched in the seacoast biosystems [25]. The prevalence of such genera could indicate anthropogenic pollutions and microbes related to plant growth promotion. Interestingly, the halophilic archaea *Haloarcula* and *Haloquadratum* were among the predominant genera in the seacoast. *Haloarcula* is an extreme halophile common in saline sediments and saltpan around the globe and is involved in denitrification [26,27]. *Haloquadratum* genus is characterized by its unique flat square shape morphology and an extended doubling time of 10 days [28].

The diversity of halophilic community was slightly higher in the coastal biosystems than in the others, but its abundance was lower. The low Shannon diversity index could be due to the sedimented nature of the coastal biosystems [29]. Sediment microbial communities have strong antagonistic properties to colonize the sediment particles and are exposed to higher concentrations of nutrients and sunlight [30]. Furthermore, coastal biosystems could also be characterized by higher salinity [31]. The relative abundance of extreme halophiles was several folds higher in the coastal biosystems than in the open sea. The predominant genus *Marinobacter* was enriched to a much greater extent than the other genera. The enrichment of *Marinobacter* could indicate anthropogenic pollution in the coastal biosystems on a global scale [32,33]. The coastal biosystems are densely populated and vulnerable to anthropogenic pollutions, climate change, and ecological degradation worldwide [34]. Such circumstances could enrich halophiles with hydrocarbon degradation characteristics.

The other dominant genera in the coastal and open sea were *Chromohalobacter, Salinibacter*, *Halorubrum*, and *Haloarcula*. Nonetheless, their abundance was substantially higher on the seacoast. These genera have also been identified in polluted hydrocarbon environments [35]. The adaptation mechanism of the identified genera involves osmoadaptation through carotenoid production, salt-in/salt-out strategy, and compatible solute through glycine Betaine/Carnitine/Choline Transporter (BCCT) [36].

### 3.2. Archaeal Extreme Halophiles Are Enriched with crtI and cruF

Halophilic microbes produce red, yellow, and orange colored carotenoid pigments. The *crtI* gene is responsible for lycopene production. The lycopene is further processed to produce α-, β-carotene, or bacterioruberin (*cruF*). The carotenoid synthesis pathways in archaeal halophiles have been reported to differ from non-halophiles by producing mostly bacterioruberin, a rare C_50_ carotenoid mainly produced by haloarchaea [4,37]. Bacterioruberin from the bacterial domain has also been reported in the literature, although in paucity, such as psychrotrophic *Arthrobacter agilis* [38], *Rhodospirillaceae* strains [39] and *Azospirillum* species [40]. *cruF* gene was observed in only 1.69% bacteria (*n* = 382/22615) and 40.75% archaea (*n* = 377/925). Despite the lower hit rate ratio of *cruF* in bacterial strains, it is quite interesting to note the presence of *cruF,* because bacterioruberin synthesis from bacterial systems has not received much attention compared to the archaeal counterpart, yet the absolute number of hits is similar. This suggests that bacterial systems could also be as prolific producers as archaea. Among the predominant extreme halophiles under bacterial domain, viz. *Marinobacter*, *Salinibacter, Halomonas,* and *Salimicrobium*, *cruF* was not detected, indicating that these genera may not produce bacterioruberin. On the contrary, extreme halophilic archaea encoded *cruF,* thus indicating potential bacterioruberin production. The predominant archaeal genus *Halorubrum* and *Haloferax* have also been reported to produce bacterioruberin [19,41,42]. The wide prevalence of *cruF* among the dominant archaeal genera strengthens the potential to harness bacterioruberin from the coastal biosystems.

### 3.3. Archaeal Halophiles Exhibit Diverse Pigmentation

Pigmented extreme halotolerant pure cultures from 410 sediments of mangroves, lakes, seashore, saltpan, estuaries, and rivers along the Arabian Sea coast (India) were screened in MSG media enforced with 3–6 M NaCl. To the best of our knowledge, this is the first attempt to isolate pigmented extreme halophilic microbes from various marine biosystems throughout the Arabian coast in this country. Seventy-seven isolates of 1351 extreme halotolerant isolates exhibited pigmentation. Despite the extremely low salinity in river sediment, the presence of pigmented halotolerant warrants further investigation of pigmented halophile diversity in freshwater bodies. This result also sheds light on carotenoid bioprospection and their derivatives from natural environments other than saline ecosystems.

Archaeal isolates exhibited yellow, orange, and red pigments, while bacterial isolates showed yellow and orange but not red pigments. Haloarcheal red pigments are mainly astaxanthin and bacterioruberin, while orange pigments could be salinixanthin [6,37]. Both pigment types have significant antimicrobial and antioxidant properties [6,19,43,44]. Yellow pigments of haloarchaea are compounds such as 4,4’-diaponeurosporene, zeaxanthin, or lutein with applications in food colorants, visual acuity, and antibacterial activity against multidrug-resistant organisms [45,46].

The predominance of yellow-pigmented isolates, especially in the seashore, manifest the occurrence of both autotrophs and heterotrophs in the marine biosystems, which require such pigments to adapt to adverse environmental conditions such as high salinity, radiation, pH, and temperature [47,48,49,50,51,52]. The predominance of yellow-pigmented isolates could also be explained by the abundance of silica and calcium in the seashore that facilitates absorption of nutrients due to the large surface area that helps develop a diverse microbial community [53].

Compared to bacterial isolates, archaeal isolates had a significant higher doubling and incubation periods for pigmentation. The prolonged incubation for carotenoid production could be because carotenoids are secondary metabolites produced in the late phase of growth. Furthermore, the process (carotenogenesis) comprises complex metabolic networks involving several enzymes, transcriptional regulatory protein, ORC1-type DNA replication protein, and GTP cyclohydrolase III that are induced mainly in the presence of high-saline conditions [26,54]. Extreme halophiles can endure high saline stress mainly through the expression of saline-resistant genes such as *rrnAC2519, cdc6A, gch3, flaC, psp A* and *rpsG* [26].

The isolates showing above 60% G + C contents were predominant in red-pigmented isolate (100%), but only in 50% and 12.5% among the orange and yellow-pigmented isolates. The red pigments were also haloarchaea members (*Haloferax* genus) resistant to saturated NaCl (6M). Previous studies have shown that G + C content is associated with genome stability in high saline and abiotic stress [55,56,57,58]. Thus, it is established that high G + C content in haloarchaeal serves as a protective measure from environmental stress, prevention of thymidine dimers and reduces UV-induced mutations [20,57]. The high G + C content is also associated with biased usage of amino acids, leading to acidic proteome [59,60], a hallmark of halophiles [57]. Acidic proteome requires a saline environment for stability, activity, and osmotic balance [61]. Such adaptation could explain the high G + C content in extreme haloarchaeal and halobacterial isolates observed in this study.

The significant enrichment of reddish and orangish pigments among the haloarchaeal group further assures the importance of biomining archaeal communities. Carotenoids and their derivatives are of particular interest because of their commercial value as food additives, colorants, and medicinal applications [62]. Global demand for carotenoid compounds is projected to be $2.0 billion by 2022 [63].

### 3.4. Biogeography of Extreme Halophiles in Arabia Sea Coast of India

Haloarchaea were solely represented by *Haloferax* species. They were recovered from all biosystems, except islands, indicating their prevalence in Arabian Sea biosystems. Interestingly, all four species of the *Haloferax* genus, i.e., *H. lucentense, H.* *sulfurifontis,* *H. chudinovii,* and *H. alexandrinus,* were isolated in Seashore sediments of Kerala. *Haloferax* species are of particular interest as they are hyper producers of bacterioruberin with high antioxidant and pharmaceutical applications [64,65,66]. *Haloferax* can also convert cheese whey/olive mill wastewater into poly (3-hydroxybutyrate-co-3-hydroxyvalerate), which shows potential applications as biodegradable biopolymer [67,68,69]. Furthermore, extreme salt tolerance is a desirable attribute for the industrial-scale production of carotenoids or whole-cell biocatalysts due to its ease in extraction, tolerance to salinity, and an array of biological applications [4,5,6,66].

The bacterial halophiles were represented by eleven genera, predominantly *Salimicrobium, Pontibacillus, Chromohalobacter, Halomonas*, and *Marinobacter*. *Salimicrobium* sp. has been reported to produce glutamate dehydrogenase, which suggests its industrial importance [70]. Extracts from *Pontibacillus* and *Chromohalobacter* have also been shown to exhibit anticancer and α-amylase activity [71,72,73,74]. Furthermore, *Chromohalobacter* degrades aromatic hydrocarbons that have a potential role in wastewater treatment and also serves as hydroxyectoine producer [75,76]. *Halomonas* species are known to synthesize ectoine and sulphate exopolysaccharides with biological activities [77,78]. Previous reports from South China Sea sediments have also isolated *Halomonas* species [79]. *Halomonas* species have also been reported to produce emulsifying agents such as P39a, which are of industrial interest [80]. Although most halophiles were isolated from Kerala sediments, several other species (such as *Virgibacillus sp.*, *Pseudomonas sp., Staphylococcus sp., Bacillus sp., Acidovorax sp*.) were only isolated from other locations. Some of these genera are of biotechnological interest: halophilic *Acidovorax* and *Staphylococcus* for instance synthetize enzymes for polyhydroxybutyrate depolymerization and thermo-tolerant alkaline lipases [81,82].

*Haloferax* dominated the mangrove and *Halomonas* the Seashore sediments. Interestingly, all isolates from Kerala biosystems were resistant to 6M NaCl. The prevalence of extreme halophiles in Kerala could be credited to several factors. Firstly, the high salinity of Arabian Sea coastal regions in western India has been well documented [83]. Furthermore, Kerala has diverse biosystems such as mangroves and brackish water interlinked by 41 freshwater rivers, leading to the formation of numerous salinity gradient estuaries. Finally, most of the halophilic species isolated from the river in the current study have been reported in the literature mainly from a saline environment [26,70]. For instance, *Virgibacillus dokdonensis* is an extracellular proteases producer isolated from Saharan Salt Lake. Similarly, *Chromohalobacter israelensis, Pontibacillus chungwhensis, Haloferax lucentense,* and *Haloferax chudinovii* have been isolated from seawater, saltpans, saline desert, etc. [26,84,85,86,87]. To the best of our knowledge, this study describes for the first time that such species are not only exclusive to the saline environment but could also inhabit freshwater sediments. The detection of such halophiles from non-saline sediments could be due to the strong enrichment during the screening step.

### 3.5. Morphological, Biochemical, and Antibiotic Resistance Profiles of Halotolerant Species

Halotolerant species inhabit harsh environmental conditions that favor diverse biochemical reactions [88]. The extremes in environmental conditions would shape and evolve their enzymes to participate in such biochemical reactions [89]. Nevertheless, salinity has a detrimental effect on enzymatic reactions [90,91]. For instance, catalase and urease activities were reported to be inversely correlated to pH and salinity [92,93,94]. However, in this study, catalase and urease activities were observed in several isolates, indicating that such enzymes in halophiles might have an evolutionary adaptation that confers tolerance to salinity to be functional [95]. Cytochrome oxidase was also highly prevalent among the extreme salt-tolerant isolates. We also observed that 66.23% of isolates were Gram-positive. Previous studies on Salt Lake, the Dead Sea, and the Wadden Sea have also observed a predominance of Gram-positive halotolerant with specific adaptions in the coastal area [96,97,98]. Furthermore, over 60% of the isolates were rod-shaped. The high proportions of rod shape halotolerant have also been observed in previous studies and could have an evolutionary advantage [97]. In line with the higher diversity of pigmentation among haloarchaea, biochemical activities were also higher in the archaeal halophiles than in the bacterial counterpart.

The landlocked nations in the northern part of India account for about 30% of the world population, which could be sewage and industrial effluent [99,100,101]. Such anthropogenic activity enriches antibiotic-resistant genes in the coastal environment [102,103,104]. Furthermore, our previous metagenomic studies identified the prevalence of ARGs in the Kerala mangrove sediments [105]. Antibiotic resistance is a global threat with a mortality of over 700,000 per year, and it is expected to reach 10 million by 2050 [106,107]. Halophiles are reported to harbor multidrug resistance through the mechanism of efflux pumps, beta-lactamase production, etc., including ARG encoded in the plasmid(s) [108,109]. In this study, halotolerant isolates had resistance to gentamycin (61.03%) and tetracycline (46.75%) at 25 µg/mL concentration. Furthermore, multidrug resistances to all four antibiotics (tetracycline, gentamicin, ampicillin, and chloramphenicol) were also observed in three isolates including RK_DM4 (*Bacillus firmus*), RK_OK1 (*Virgibacillus dokdonensis*), and RK_OK3 (*Staphylococcus saprophyticus*). In line with this, our previous study on multidrug-resistant (MDR) bacteria in mangrove sediments from the Arabian coast, India, has also identified *Bacillus firmus* as resistant to all four antibiotics [29]. *Bacillus firmus* and *Staphylococcus saprophyticus* promote plant growth and degrade hydrocarbon, respectively [110,111,112]. However, they are also well-known as causative agents for food spoilage and urinary tract infections [113,114]. Antibiotic resistance is of grave concern owing to its rapid spread through horizontal gene transfer [115]. The prevalence of MDR isolates observed in this study indicates the potential of antibiotic-resistant gene (ARG) horizontal transfer from environmental to clinically relevant pathogens [116,117,118].

## 4. Materials and Methods

### 4.1. Metagenomic Analysis

Whole metagenome datasets (*n* = 209; ~1.1 Terabyte) of open sea and coastal region were retrieved from the MG-RAST (Metagenomic Rapid Annotations using Subsystems Technology) repository server (Figure 7).

The metagenome datasets originate from various locations such as the Atlantic Ocean, Indian Ocean, Southern Ocean, Pacific Ocean, coastline region of Russia, Australia, Egypt, Pakistan, Mexico, and Antarctica. (Table S4). All raw datasets were processed in the MG-RAST pipeline to avoid differences in the in silico approach. The pipeline includes removing low-quality sequence reads based on the Phred score (less than 20), extracting duplicate reads and host DNA such as human, and then processing for the taxonomic identification and functional profiling. The OUT matrix was batch-normalized with preprocessCore v1.52.0 [119], and the microbiome data were analyzed using phyloseq v1.34.0 [120] and microbiome v1.12.0 package in R v4.0.3 (R Core Team 2020) [121]. We retrieved 23,540 whole-genome CDS profiles from the EzBioCloud database [122] by selecting a single species per genus. In-house R scripts were used to detect the presence or absence of *crtI*, *crtL*, *cruF*, and *crtD* genes from all CDS profiles. These are key genes involved in the carotenogenesis pathway; thus, *crtD* codes for carotenoid 3,4-desaturase; *crtI* codes for lycopene-forming enzyme; *crtL* encodes lycopene beta cyclase and *cruF* codes for bisanhydrobacterioruberin hydratase [2].

### 4.2. Sediment Sampling

A total of 410 locations along the Arabian Sea coastal area between Gujarat (Latitude 22°27′34.7” N Longitudes; 69°04′01.8” E) and Kanyakumari (Latitude; 8°05′11.8” N Longitudes; 77°33′16.0” E) (India) (Table S5) were surveyed for the sampling of coastal region sediment. Triplicate sediments were collected in individual sterile plastic bags using a sterile spatula from the upper 5–10 cm during June–November, 2016–2017. The sediment samples were preserved in ice and transported to the lab.

### 4.3. Enrichment and Isolation of Pigmented Halophiles

The pigmented halophiles were enriched from the sediments in Modified Sehgal and Gibbon’s (MSG) media (Table 2) by incubating at 37 °C in a rotary shaking incubator at 100 rpm for 14 days or until pigmentation was observed. The enriched broth was subsequently used as inoculum (10 µL) in modified MSG agar plates [26] augmented with 3 M, 4 M, 5 M, and 6 M NaCl. The plates were further incubated at 37 °C until red, yellow, or orange colonies appeared. The colonies were sub-cultured several times to ascertain the purity, and stereomicroscopic images were obtained using Leica S8 APO with Leica MC 170 HD camera. Pure cultures were stored in MSG slants and glycerol stocks at −80 °C.

### 4.4. 16S rRNA Gene Identification and Phylogenetic Analysis

Genomic DNA was extracted using HiPurA™ Bacterial Genomic DNA Purification Kit (HiMedia, India). The 16S rRNA gene sequence of archaeal was amplified using Arch344F and Arch915R, while the bacterial 16S rRNA gene was amplified with 8F and 518R (Table 3) [123,124].

The raw sequences were subjected to quality control by trimming off the low-quality reads, and a similarity match was determined using the NCBI BLAST tool (8600 Rockville PikeBethesda, MD 20,894, USA). A percentage similar to 97% or higher was considered as the same species [125]. Sequences were deposited in the NCBI gene bank with accession number MT322457-MT322533. Sequence alignment and phylogenetic tree construction were performed using MEGA v7.0 software (Arizona State University and Masatoshi Nei, Pennsylvania State University) with the Neighbor-joining algorithms based on the Tamura 3-parameter method with 1000 bootstrap [126].

### 4.5. Optimization of NaCl Concentration on Growth and Pigment Production

Optimization of growth and pigment production was performed in MSG broth augmented with 3 M and 6 M NaCl at 37 °C for 14 days [26]. Total carotenoid production was determined by culturing in 6 M NaCl MSG broth and absorbance monitored at 490 nm with 12 h intervals for 14 days using Epoch Microplate Spectrophotometer (BioTek Instruments, Inc., Winooski, VT, USA). Pigment production was further confirmed by streaking the broth on MSG plates with the respective NaCl concentration at 37 °C for 14 days.

### 4.6. Morphological and Biochemical Analysis

All isolates were cultivated at the respective optimum growth condition for biochemical assays. The isolates were stained using the standard Gram Method microscopic observation. The motility by organism was determined with semi-solid agar method by inoculating it in a semi-solid MSG medium and incubating for 14 days. Catalase activity was determined using 3% (*v/v*) hydrogen peroxide, and cytochrome oxidases activity was assessed by spotting log phase culture on Whatman No. 1 filter paper followed by adding a few drops of oxidase reagent (Himedia, India) [127,128]. For rapid confirmation of phenotypic characterization, API 20E strips (BioMerieux, Durham, NC, USA) were implemented according to the manufacturer’s protocol [129]. The API 20E detection system (BioMerieux, Durham, NC, USA) is an elongated panel with several units containing dehydrated substrates that can be inoculated with a log-phase bacterial suspension. The biochemical tests investigated with the API 20E system are β-galactosidase (ONPG), arginine dihydrolase (ADH), lysine decarboxylase (LDC), ornithine decarboxylase (ODC), citrate utilization (CIT), H_2_S production (H2S), urease (URE), tryptophan deaminase (TDA), indole production (IND), Voges–Proskauer (VP), gelatinase (GEL), glucose (GLU), mannitol (MAN), inositol (INO), sorbitol (SOR), rhamnose (RHA), saccharose (SAC), melibiose (MEL), amygdalin (AMY), arabinose (ARA), xylose (XYL), esculin (ESC), lactose (LAC), mannose (MNE), salicin (SAL), glycerol (GLY), cellobiose (CEL), melezitose (MLZ), raffinose (RAF), and trehalose (TRE).

### 4.7. G + C Content Estimation

Genomic DNA was normalized to 5 µg/µL in nuclease-free water and diluted 1:100,000 with SYBR Green (Origin, Kerala, India). Melting curve genotyping was performed using Roche Light Cycler 480 II (Roche) RT PCR system. The T_m_ values were calculated from the minimum value of the slope tangent to the melting curve of DNA versus temperature. G + C% was plotted against the calibration curve derived from the whole-genome sequence of *Pseudomonas aeruginosa* PAO1 and *E. coli* K12 [130].

### 4.8. Antibiotics Resistance Profiling

Antibiotic resistance profiles of all the isolates were evaluated by the serial dilution method. MSG agar plates were supplemented with 25, 50, and 100 μg/mL of ampicillin, gentamicin, chloramphenicol, and tetracycline, individually for each antibiotic.

### 4.9. Statistical Analysis

The statistically significant difference between the groups was determined by the Kruskal–Wallis test using PAST (PAleontological STatistics) v3.26 software [131]. The difference in G + C content between domains was analyzed with Epps-singleton. A *p*-value of less than 0.05 was considered statistically significant. Principal Component Analysis (PCA) was generated using ClustVis [132].

## 5. Conclusions

Differences between biotic and abiotic components of the open sea and coastal regions profoundly regulates the microbial community in these biosystems. However, there is a paucity in comparative studies on microbial biodiversity between these ecosystems. In this study, we implemented a whole-genome meta-analysis of the shotgun metagenome and attempted to isolate extreme halophiles from the coastal region. Shotgun metagenomic analysis of the global open sea and seacoast biosystem revealed diverse and abundant halophilic microbes in the seacoast biosystem. The identification of halophilic archaea *Haloarcula* and *Haloquandratum* as signature-predominant genera in the coastal biosystems and also the significant (*p* < 0.05) enrichment of halophilic community (*p* < 0.05) in the coastal regions compared to the open sea provides a strong hit that coastal biosystems could be a reservoir of archaeal halophiles. Whole-genome analysis revealed that *cruF* gene was present in 40.75% of Archaea (*n* = 377/925) but only in 1.69% of bacterial genomes (*n* = 382/22,615). However, attempts to isolate halophiles from the coastal sediments of the Arabian coastline through cultivable techniques indicated dominance of the halophilic *Haloferax* genus. This suggested the disparity between culture-dependent and -independent techniques and indicates the need for the development of robust culture media/techniques. Carotenoid pigment production by the pure culture (*n* = 1351) revealed that all red-pigmented isolates were represented exclusively under the *Haloferax* genus. The halophiles were also multidrug-resistant to tetracycline, gentamicin, ampicillin, and chloramphenicol. Our study shows that bacterioruberin carotenoids are not only exclusive to the archaeal domain, but the bacterial domain could also be a reservoir of bacterioruberin derivatives. Nevertheless, the predominance of archaeal *Haloferax* in coastal biosystems and its extremophilic parameters such as high G + C content (>60%), NaCl tolerance (6 M), and bacterioruberin (red pigments) suggest the crucial roles of haloarchaea in coastal niches and potential implications in pharmaceuticals and biotechnological industry.

## Figures and Tables

**Table 1:** Carotenoid gene abundances in bacterial and archaeal strains based on whole-genome sequences.

Domain	Bacteria				Archaea			
No. of strains	22,615				925			
*crt* genes	*crtI*	*crtL*	*cruF*	*crtD*	*crtI*	*crtL*	*crtD*	*cruF*
No. of genes	14,795	3001	397	1837	427	0	568	415
No. of unique strains	6766	2684	382	1643	292	0	329	377

**Table 2:** Media composition of Modified Sehgal and Gibbon’s (MSG) used to isolate and enrich halophiles.

Serial Number	Ingredients	g/L or Molar Concentration
1	NaCl	1–6 M
2	Tryptone	5
3	Yeast extract	10
4	Potassium chloride	2
5	Trisodium citrate	3
6	MgSO_4_	20

**Table 3:** 16S rRNA gene universal primer used for identification of the archaeal and bacteria extreme halophiles.

Primer Name	Universal Primer	Sequence
Arch344F	Archeal Forward	5′-ACGGGGTGCAGGCGCGA-3′
Arch915R	Archeal Reverse	5′-GTGCTCCCCCGCCAATTCCT-3′
8F	Bacterial Forward	5′-AGAGTTTGATCCTGGCTCAG-3′
518R	Bacterial Reverse	5′-ATTACCGCGGCTGCTGG-3′

## Data Availability

The 16S rRNA gene sequences generated in this investigation are available NCBI gene bank under the accession number MT322457–MT322533.

## Supplementary Materials

The following are available online at https://www.mdpi.com/article/10.3390/md19080442/s1. Figure S1: Pigmentation in the extreme halophiles grouped according to (a) pigmentation color, (b) biosystems, and (c) geography. (d) Antibiotic resistance profiles of the halotolerant isolates. Figure S2: Distribution of halophilic isolates resistant to (a) 6M and (b) 3M NaCl enforced in MSG media. (c) 3M and 6M NaCl resistance isolated based on pigmentation. Most of the yellow pigmented isolates were resistant only up to 3M NaCl. The G+C content of extreme halotolerant (d) archaea and (e) bacteria. Pigmentation production at various time points in 6M NaCl by halophilic (f) archaeal and (g) bacterial isolates. Figure S3: Phylogenetic tree, constructed using the neighbor-joining method, of halophilic (a) archaea and (b) bacteria isolated from Kerala coastal region, (c) bacterial halophiles from Gujarat, Maharashtra, Goa, Karnataka, and Lakshadweep coastal regions. Table S1: Gram nature, motility, aerobic and pH parameters of the isolated halophiles. Table S2: Biochemical analysis of the microbial isolates: Catalase, Oxidases, urease (URE), gelatinase (GEL), glucose (GLU), arabinose (ARA), amygdalin (AMY), xylose (XYL), esculin (ESC), saccharose (SAC), melibiose (MEL), lactose (LAC), mannose (MNE), β-galactosidase (ONPG), arginine dihydrolase (ADH), lysine decarboxylase (LDC), ornithine decarboxylase (ODC), citrate utilization (CIT), H2S production (H2S), tryptophane deaminase (TDA), indole production (IND), Voges–Proskauer (VP), mannitol (MAN), inositol (INO), sorbitol (SOR), rhamnose (RHA), salicin (SAL), glycerol (GLY), cellobiose (CEL), melezitose (MLZ), raffinose (RAF), and trehalose (TRE). Table S3: 16S rRNA gene sequence similarity of halotolerant microbial isolates with location and accession number. Table S4: The metagenome data set was retrieved from the MG-RAST server for the comparative analysis of the open sea and coastal microbiome. Sampling with a distance of 100+ km, calculated manually with https://www.freemaptools.com/measure-distance.htm, away from coastline was considered as open sea. Table S5: Sampling locations (*n* = 410) across the Arabian seacoast (India) from seven biosystems. Script: The R script for checking the presence of required gene.

## References

[1] Calegari-Santos R., Diogo R.A., Fontana J.D., Bonfim T.M.B. (2016). Carotenoid Production by Halophilic Archaea Under Different Culture Conditions. Curr. Microbiol..

[2] Moopantakath J., Imchen M., Siddhardha B., Kumavath R. (2020). 16s rRNA metagenomic analysis reveals predominance of Crtl and CruF genes in Arabian Sea coast of India. Sci. Total Environ..

[3] Kirti K., Amita S., Priti S., Kumar A.M., Jyoti S. (2014). Colorful World of Microbes: Carotenoids and Their Applications. Adv. Biol..

[4] Rodrigo-Baños M., Garbayo I., Vílchez C., Bonete M.-J., Martínez-Espinosa R.M. (2015). Carotenoids from Haloarchaea and Their Potential in Biotechnology. Mar. Drugs.

[5] Giani M., Garbayo I., Vílchez C., Martínez-Espinosa R.M. (2019). Haloarchaeal Carotenoids: Healthy Novel Compounds from Extreme Environments. Mar. Drugs.

[6] Hegazy G., Abu-Serie M.M., Abo-Elela G.M., Ghozlan H., Sabry S.A., Soliman N.A., Abdel-Fattah Y.R. (2020). In vitro dual (anticancer and antiviral) activity of the carotenoids produced by haloalkaliphilic archaeon Natrialba sp. M6. Sci. Rep..

[7] Oren A. (2015). Halophilic microbial communities and their environments. Curr. Opin. Biotechnol..

[8] Edgcomb V.P., Biddle J.F., Edgcomb V.P.A., Altenbach A.V., Bernhard J.M., Seckbach J. (2012). Cellular Origin, Life in Extreme Habitats and Astrobiology.

[9] Jensen M.W., Matlock S.A., Reinheimer C.H., Lawlor C.J., Reinheimer T.A., Gorrell A. (2015). Potassium stress growth characteristics and energetics in the haloarchaeon Haloarcula marismortui. Extremophiles.

[10] Hallsworth J.E. (2019). Microbial unknowns at the saline limits for life. Nat. Ecol. Evol..

[11] Steinmuller H.E., Foster T.E., Boudreau P., Hinkle C.R., Chambers L.G. (2020). Characterization of herbaceous encroachment on soil biogeochemical cycling within a coastal marsh. Sci. Total Environ..

[12] Dang H., Lovell C.R. (2016). Microbial Surface Colonization and Biofilm Development in Marine Environments. Microbiol. Mol. Biol. Rev..

[13] Toulza E., Tagliabue A., Blain S., Piganeau G. (2012). Analysis of the Global Ocean Sampling (GOS) Project for Trends in Iron Uptake by Surface Ocean Microbes. PLoS ONE.

[14] De la Calle F. (2017). Marine microbiome as source of natural products. Microb. Biotechnol..

[15] Mena C., Reglero P., Balbín R., Martín M., Santiago R., Sintes E. (2020). Seasonal Niche Partitioning of Surface Temperate Open Ocean Prokaryotic Communities. Front. Microbiol..

[16] Moran M.A. (2015). The global ocean microbiome. Science.

[17] De Carvalho C.C.C.R., Caramujo M.J. (2017). Carotenoids in Aquatic Ecosystems and Aquaculture: A Colorful Business with Implications for Human Health. Front. Mar. Sci..

[18] Giani M., Martínez-Espinosa R. (2020). Carotenoids as a Protection Mechanism against Oxidative Stress. Haloferax Mediterr. Antioxid..

[19] Giani M., Montero-Lobato Z., Garbayo I., Vílchez C., Vega J., Martínez-Espinosa R. (2021). *Haloferax mediterranei* Cells as C50 Carotenoid Factories. Mar. Drugs.

[20] Paul S., Bag S.K., Das S., Harvill E., Dutta C. (2008). Molecular signature of hypersaline adaptation: Insights from genome and proteome composition of halophilic prokaryotes. Genome Biol..

[21] Hartman A.L., Norais C., Badger J.H., Delmas S., Haldenby S., Madupu R., Robinson J., Khouri H., Ren Q., Lowe T.M. (2010). The Complete Genome Sequence of Haloferax volcanii DS2, a Model Archaeon. PLoS ONE.

[22] Smith D.P., Nicora C.D., Carini P., Lipton M.S., Norbeck A.D., Smith R.D., Giovannoni S.J. (2016). Proteome Remodeling in Response to Sulfur Limitation in “Candidatus Pelagibacter ubique. ” mSystems.

[23] Kasai Y., Kishira H., Sasaki T., Syutsubo K., Watanabe K., Harayama S. (2002). Predominant growth of Alcanivorax strains in oil-contaminated and nutrient-supplemented sea water. Environ. Microbiol..

[24] Chernikova T.N., Bargiela R., Toshchakov S.V., Shivaraman V., Lunev E.A., Yakimov M.M., Thomas D.N., Golyshin P.N. (2020). Hydrocarbon-Degrading Bacteria Alcanivorax and Marinobacter Associated with Microalgae Pavlova lutheri and Nannochloropsis oculata. Front. Microbiol..

[25] Astriani M., Zubaidah S., Abadi A.L., Suarsini E. (2020). Pseudomonas plecoglossicida as a novel bacterium for phosphate solubilizing and indole-3-acetic acid-producing from soybean rhizospheric soils of East Java, Indonesia. Biodiversitas J. Biol. Divers..

[26] Thombre R.S., Shinde V.D., Oke R.S., Dhar S.K., Shouche Y.S. (2016). Biology and survival of extremely halophilic archaeon Haloarcula marismortui RR12 isolated from Mumbai salterns, India in response to salinity stress. Sci. Rep..

[27] Bonete M.-J., Martínez-Espinosa R.M., Pire C., Zafrilla B., Richardson D.J. (2008). Nitrogen metabolism in haloarchaea. Saline Syst..

[28] Bolhuis H., Martín-Cuadrado A.B., Rosselli R., Pasic L., Rodriguez-Valera F. (2017). Transcriptome analysis of Haloquadratum walsbyi: Vanity is but the surface. BMC Genom..

[29] Imchen M., Vennapu R.K., Ghosh P., Kumavath R. (2019). Insights into Antagonistic Interactions of Multidrug Resistant Bacteria in Mangrove Sediments from the South Indian State of Kerala. Microorgasims.

[30] Ask J., Rowe O., Brugel S., Strömgren M., Byström P., Andersson A. (2016). Importance of coastal primary production in the northern Baltic Sea. Ambio.

[31] Baldwin A.H.A., Mendelssohn I. (1998). Effects of salinity and water level on coastal marshes: An experimental test of disturbance as a catalyst for vegetation change. Aquat. Bot..

[32] Xu X., Liu W., Tian S., Wang W., Qi Q., Jiang P., Gao X., Li F., Li H., Yu H. (2018). Petroleum Hydrocarbon-Degrading Bacteria for the Remediation of Oil Pollution Under Aerobic Conditions: A Perspective Analysis. Front. Microbiol..

[33] Jamal M.T. (2020). Enrichment of Potential Halophilic Marinobacter Consortium for Mineralization of Petroleum Hydrocarbons and Also as Oil Reservoir Indicator in Red Sea, Saudi Arabia. Polycycl. Aromat. Compd..

[34] Lu Y., Yuan J., Lu X., Su C., Zhang Y., Wang C., Cao X., Li Q., Su J., Ittekkot V. (2018). Major threats of pollution and climate change to global coastal ecosystems and enhanced management for sustainability. Environ. Pollut..

[35] Fathepure B.Z. (2014). Recent studies in microbial degradation of petroleum hydrocarbons in hypersaline environments. Front. Microbiol..

[36] Vargas C., Argandona M., Reina-Bueno M., Rodriguez-Moya J., Fernandez-Aunion C., Nieto J.J. (2008). Unravelling the adaptation responses to osmotic and temperature stress in *Chromohalobacter salexigens*, a bacterium with broad salinity tolerance. Saline Syst..

[37] Giani M., Miralles-Robledillo J.M., Peiró G., Pire C., Martínez-Espinosa R.M. (2020). Deciphering Pathways for Carotenogenesis in Haloarchaea. Molecules.

[38] Fong N.J.C., Burgess M.L., Barrow K.D., Glenn D.R. (2001). Carotenoid accumulation in the psychrotrophic bacterium *Arthrobacter agilis* in response to thermal and salt stress. Appl. Microbiol. Biotechnol..

[39] Rai A.K., Dubey A.P., Kumar S., Dutta D., Mishra M.N., Singh B.N., Tripathi A.K. (2016). Carotenoid Biosynthetic Pathways Are Regulated by a Network of Multiple Cascades of Alternative Sigma Factors in Azospirillum brasilense Sp7. J. Bacteriol..

[40] Mishra S., Chanotiya C.S., Shanker K., Tripathi A.K. (2021). Characterization of carotenoids and genes encoding their biosynthetic pathways in *Azospirillum brasilense*. FEMS Microbiol. Lett..

[41] Naziri D., Hamidi M., Hassanzadeh S., Tarhriz V., Zanjani B.M., Nazemyieh H., Hejazi M.A., Hejazi M.S. (2013). Analysis of Carotenoid Production by Halorubrum sp. TBZ126; an Extremely Halophilic Archeon from Urmia Lake. Adv. Pharm. Bull..

[42] Zalazar L., Pagola P., Miró M.V., Churio M.S., Cerletti M., Martínez C., Iniesta-Cuerda M., Soler A., Cesari A., De Castro R. (2018). Bacterioruberin extracts from a genetically modified hyperpigmented Haloferax volcanii strain: Antioxidant activity and bioactive properties on sperm cells. J. Appl. Microbiol..

[43] Ambati R.R., Phang S.-M., Ravi S., Aswathanarayana R.G. (2014). Astaxanthin: Sources, Extraction, Stability, Biological Activities and Its Commercial Applications—A Review. Mar. Drugs.

[44] Hou J., Cui H.-L. (2017). In Vitro Antioxidant, Antihemolytic, and Anticancer Activity of the Carotenoids from Halophilic Archaea. Curr. Microbiol..

[45] Bernstein P.S., Li B., Vachali P.P., Gorusupudi A., Shyam R., Henriksen B.S., Nolan J.M. (2016). Lutein, zeaxanthin, and meso-zeaxanthin: The basic and clinical science underlying carotenoid-based nutritional interventions against ocular disease. Prog. Retin. Eye Res..

[46] Sibero M.T., Igarashi Y., Radjasa O.K., Sabdono A., Trianto A., Zilda D.S., Wijaya Y.J. (2019). Sponge-associated fungi from a mangrove habitat in Indonesia: Species composition, antimicrobial activity, enzyme screening and bioactive profiling. Int. Aquat. Res..

[47] Estrada M., Henriksen P., Gasol J.M.O., Casamayor E., Pedrãs-Aliã C. (2004). Diversity of planktonic photoautotrophic microorganisms along a salinity gradient as depicted by microscopy, flow cytometry, pigment analysis and DNA-based methods. FEMS Microbiol. Ecol..

[48] Amoozegar M.A., Makhdoumi A., Mehrshad M., Fazeli S.A.S., Ventosa A. (2013). Halopenitus malekzadehii sp. nov., an extremely halophilic archaeon isolated from a salt lake. Int. J. Syst. Evol. Microbiol..

[49] Repeta D.J., Gagosian R.B. (1982). Carotenoid transformations in coastal marine waters. Nat. Cell Biol..

[50] Leiva S., Alvarado P., Huang Y., Wang J., Garrido I. (2015). Diversity of pigmented Gram-positive bacteria associated with marine macroalgae from Antarctica. FEMS Microbiol. Lett..

[51] Kim J.-G., Gwak J.-H., Jung M.-Y., An S.-U., Hyun J.-H., Kang S., Rhee S.-K. (2019). Distinct temporal dynamics of planktonic archaeal and bacterial assemblages in the bays of the Yellow Sea. PLoS ONE.

[52] Khan S.A., Vahed S.Z., Forouhandeh H., Tarhriz V., Chaparzadeh N., Hejazi M.A., Jeon C.O., Hejazi M.S. (2020). *Halomonas urmiana* sp. nov., a moderately halophilic bacterium isolated from Urmia Lake in Iran. Int. J. Syst. Evol. Microbiol..

[53] Whitman R.L., Harwood V.J., Edge T.A., Nevers M.B., Byappanahalli M.N., Vijayavel K., Brandão J., Sadowsky M.J., Alm E.W., Crowe A. (2014). Microbes in beach sands: Integrating environment, ecology and public health. Rev. Environ. Sci. Bio/Technology.

[54] D’Souza S.E., Altekar W. (1997). Adaptive response of Haloferax mediterranei to low concentrations of NaCl (<20%) in the growth medium. Arch. Microbiol..

[55] Šmarda P., Bureš P., Horová L., Leitch I.J., Mucina L., Pacini E., Tichý L., Grulich V., Rotreklová O. (2014). Ecological and evolutionary significance of genomic GC content diversity in monocots. Proc. Natl. Acad. Sci. USA.

[56] Yakovchuk P., Protozanova E., Frank-Kamenetskii M.D. (2006). Base-stacking and base-pairing contributions into thermal stability of the DNA double helix. Nucleic Acids Res..

[57] Jones D.L., Baxter B.K. (2016). Bipyrimidine Signatures as a Photoprotective Genome Strategy in G + C-rich *Halophilic archaea*. Life.

[58] Sato Y., Kimura H. (2019). Temperature-dependent expression of different guanine-plus-cytosine content 16S rRNA genes in Haloarcula strains of the class Halobacteria. Antonie Leeuwenhoek.

[59] Palidwor G.A., Perkins T.J., Xia X. (2010). A General Model of Codon Bias Due to GC Mutational Bias. PLoS ONE.

[60] Du M.-Z., Liu S., Zeng Z., Alemayehu L.A., Wei W., Guo F.-B. (2018). Amino acid compositions contribute to the proteins’ evolution under the influence of their abundances and genomic GC content. Sci. Rep..

[61] Eoren A. (2013). Life at high salt concentrations, intracellular KCl concentrations, and acidic proteomes. Front. Microbiol..

[62] Bogacz-Radomska L., Harasym J., Piwowar A. (2020). Commercialization aspects of carotenoids. Carotenoids: Properties, Processing and Applications.

[63] Liu L., Chen J. (2019). Systems and Synthetic Biotechnology for Production of Nutraceuticals.

[64] Chen C.W., Hsu S.-H., Lin M.-T., Hsu Y.-H. (2015). Mass production of C50 carotenoids by *Haloferax mediterranei* in using extruded rice bran and starch under optimal conductivity of brined medium. Bioprocess Biosyst. Eng..

[65] Kumar S., Grewal J., Sadaf A., Hemamalini R., Khare S.K. (2016). Halophiles as a source of polyextremophilic α-amylase for industrial applications. AIMS Microbiol..

[66] Haque R.U., Paradisi F., Allers T. (2019). *Haloferax volcanii* as immobilised whole cell biocatalyst: New applications for halophilic systems. Appl. Microbiol. Biotechnol..

[67] Pais J., Serafim L., Freitas F., Reis M.A. (2016). Conversion of cheese whey into poly(3-hydroxybutyrate-co-3-hydroxyvalerate) by Haloferax mediterranei. New Biotechnol..

[68] Alsafadi D., Al-Mashaqbeh O. (2017). A one-stage cultivation process for the production of poly-3-(hydroxybutyrate-co-hydroxyvalerate) from olive mill wastewater by *Haloferax mediterranei*. New Biotechnol..

[69] Simó-Cabrera L., García-Chumillas S., Hagagy N., Saddiq A., Tag H., Selim S., AbdElgawad H., Agüero A.A., Sánchez F.M., Cánovas V. (2021). Haloarchaea as Cell Factories to Produce Bioplastics. Mar. Drugs.

[70] Li X., Yu H.-Y. (2012). Purification and characterization of novel organic-solvent-tolerant β-amylase and serine protease from a newly isolated Salimicrobium halophilum strain LY20. FEMS Microbiol. Lett..

[71] Sagar S., Esau L., Holtermann K., Hikmawan T., Zhang G., Stingl U., Bajic V.B., Kaur M. (2013). Induction of apoptosis in cancer cell lines by the Red Sea brine pool bacterial extracts. BMC Complement. Altern. Med..

[72] Choi E.J., Nam S.-J., Paul L., Beatty D., Kauffman C., Jensen P.R., Fenical W. (2015). Previously Uncultured Marine Bacteria Linked to Novel Alkaloid Production. Chem. Biol..

[73] Esau L., Zhang G., Sagar S., Stingl U., Bajic V.B., Kaur M. (2019). Mining the deep Red-Sea brine pool microbial community for anticancer therapeutics. BMC Complement. Altern. Med..

[74] Fang W., Xue S., Deng P., Zhang X., Wang X., Xiao Y., Fang Z. (2019). AmyZ1: A novel α-amylase from marine bacterium Pontibacillus sp. ZY with high activity toward raw starches. Biotechnol. Biofuels.

[75] Rodríguez-Moya J., Argandoña M., Guerra F.I., Nieto J.J., Vargas C. (2013). Temperature- and Salinity-Decoupled Overproduction of Hydroxyectoine by Chromohalobacter salexigens. Appl. Environ. Microbiol..

[76] Erdogmus S.F., Korcan S.E., Konuk M., Guven K.M.B.M. (2015). Aromatic Hydrocarbon Utilization Ability of *Chromohalobacter* sp.. Ekoloji.

[77] Llamas I., Béjar V., Martínez-Checa F., Martínez-Cánovas M.J., Molina I., Quesada E. (2011). *Halomonas stenophila* sp. nov., a halophilic bacterium that produces sulphate exopolysaccharides with biological activity. Int. J. Syst. Evol. Microbiol..

[78] Haque R.U., Paradisi F., Allers T. (2019). Haloferax volcanii for biotechnology applications: Challenges, current state and perspectives. Appl. Microbiol. Biotechnol..

[79] Long M.-R., Zhang D.-F., Yang X.-Y., Zhang X.-M., Zhang Y.-G., Zhang Y.-M., Zhu H., Li W.-J. (2013). Halomonas nanhaiensis sp. nov., a halophilic bacterium isolated from a sediment sample from the South China Sea. Antonie Leeuwenhoek.

[80] Gutierrez T., Morris G., Ellis D., Mulloy B., Aitken M.D. (2019). Production and characterisation of a marine Halomonas surface-active exopolymer. Appl. Microbiol. Biotechnol..

[81] Chauhan M., Garlapati V.K. (2013). Production and Characterization of a Halo-, Solvent-, Thermo-tolerant Alkaline Lipase by Staphylococcus arlettae JPBW-1, Isolated from Rock Salt Mine. Appl. Biochem. Biotechnol..

[82] Egusa E.A., Edwards D.J., Thao M.L., Kirk L.L., Hanne L.F. (2018). Isolation and Characterization of Bacteria that Produce Polyhydroxybutyrate Depolymerases†. J. Microbiol. Biol. Educ..

[83] Kumar S.P., Prasad T.G. (1999). Formation and spreading of Arabian Sea high-salinity water mass. J. Geophys. Res. Space Phys..

[84] Arahal D.R., García M.T., Ludwig W., Schleifer K.H., Ventosa A. (2001). Transfer of *Halomonas canadensis* and *Halomonas israelensis* to the genus Chromohalobacter as *Chromohalobacter canadensis* comb. nov. and *Chromohalobacter israelensis* comb. nov. Int. J. Syst. Evol. Microbiol..

[85] Gutierrez C.M., Kamekura M., Holmes M.L., Dyall-Smith M.L., Ventosa A. (2002). Taxonomic characterization of Haloferax sp. (“H. alicantei“) strain Aa 2.2: Description of *Haloferax lucentensis* sp. nov. Extremophiles.

[86] Lim J.-M., Jeon C.O., Song S.M., Kim C.-J. (2005). *Pontibacillus chungwhensis* gen. nov., sp. nov., a moderately halophilic Gram-positive bacterium from a solar saltern in Korea. Int. J. Syst. Evol. Microbiol..

[87] Saralov A.I., Baslerov R.V., Kuznetsov B.B. (2013). *Haloferax chudinovii* sp. nov., a halophilic archaeon from *Permian potassium* salt deposits. Extremophiles.

[88] DasSarma S., DasSarma P., Laye V.J., Schwieterman E.W. (2020). Extremophilic models for astrobiology: Haloarchaeal survival strategies and pigments for remote sensing. Extremophiles.

[89] Pratap R., Geetanjali S., Indresh M., Maurya K., Wei Y., Singh R.P., Manchanda G., Maurya I.K., Wei Y. (2020). Microbial Versatility in Varied Environments.

[90] Xie X., Pu L., Wang Q., Zhu M., Xu Y., Zhang M. (2017). Response of soil physicochemical properties and enzyme activities to long-term reclamation of coastal saline soil, Eastern China. Sci. Total Environ..

[91] Indira D., Das B., Balasubramanian P., Jayabalan R. (2018). Sea Water as a Reaction Medium for Bioethanol Production. Microbial Biotechnology.

[92] Pan C., Liu C., Zhao H., Wang Y. (2013). Changes of soil physico-chemical properties and enzyme activities in relation to grassland salinization. Eur. J. Soil Biol..

[93] Mouhamad R.S., Razaq I.B., Fadhel A.S., Yousir S.A., Taha D.I., Iqbal M. (2014). Urease Activity under Salinity Stress in Calcareous Soils of Semi-Arid Regions of Iraq. Int. J. Chem. Biochem. Sci..

[94] Yang H., Hu J., Long X., Liu Z., Rengel Z. (2016). Salinity altered root distribution and increased diversity of bacterial communities in the rhizosphere soil of Jerusalem artichoke. Sci. Rep..

[95] Arai S., Shibazaki C., Shimizu R., Adachi M., Ishibashi M., Tokunaga H., Tokunaga M. (2020). Catalytic mechanism and evolutionary characteristics of thioredoxin from *Halobacterium salinarum* NRC-1. Acta Crystallogr. Sect. D Struct. Biol..

[96] Ventosa A., Nieto J.J., Oren A. (1998). Biology of Moderately Halophilic Aerobic Bacteria. Microbiol. Mol. Biol. Rev..

[97] Barbosa D.C., Von Der Weid I., Vaisman N., Seldin L. (2006). Halotolerant Spore-Forming Gram-Positive Bacterial Diversity Associated with *Blutaparon portulacoides* (St. Hill.) Mears, a Pioneer Species in Brazilian Coastal Dunes. J. Microbiol. Biotechnol..

[98] Stevens H., Brinkhoff T., Rink B., Vollmers J., Simon M. (2007). Diversity and abundance of Gram positive bacteria in a tidal flat ecosystem. Environ. Microbiol..

[99] Islam S., Tanaka M. (2004). Impacts of pollution on coastal and marine ecosystems including coastal and marine fisheries and approach for management: A review and synthesis. Mar. Pollut. Bull..

[100] Keesing J., Irvine T. (2020). Coastal Biodiversity in the Indian Ocean: The Known, the Unknown and the Unknowable. Int. J. Mol. Sci..

[101] Wafar M., Venkataraman K., Ingole B., Khan S.A., LokaBharathi P. (2011). State of Knowledge of Coastal and Marine Biodiversity of Indian Ocean Countries. PLoS ONE.

[102] Chen Q., An X., Li H., Su J., Ma Y., Zhu Y.-G. (2016). Long-term field application of sewage sludge increases the abundance of antibiotic resistance genes in soil. Environ. Int..

[103] Fresia P., Antelo V., Salazar C., Giménez M., D’Alessandro B., Afshinnekoo E., Mason C., Gonnet G.H., Iraola G. (2019). Urban Metagenomics Uncover Antibiotic Resistance Reservoirs in Coastal Beach and Sewage Waters. Microbiome.

[104] Griffin D.W., Banks K., Gregg K., Shedler S., Walker B.K. (2020). Antibiotic Resistance in Marine Microbial Communities Proximal to a Florida Sewage Outfall System. Antibiotics.

[105] Imchen M., Kumavath R., Barh D., Vaz A., Góes-Neto A., Tiwari S., Ghosh P., Wattam A.R., Azevedo V. (2018). Comparative mangrove metagenome reveals global prevalence of heavy metals and antibiotic resistome across different ecosystems. Sci. Rep..

[106] Assar A., Abdelraoof M.I., Abdel-Maboud M., Shaker K.H., Menshawy A., Swelam A.H., Eid M., Khalid R., Mogahed M., Abushouk A.I. (2020). Knowledge, attitudes, and practices of Egypt’s future physicians towards antimicrobial resistance (KAP-AMR study): A multicenter cross-sectional study. Environ. Sci. Pollut. Res..

[107] Hermsen E.D., MacGeorge E.L., Andresen M.-L., Myers L.M., Lillis C.J., Rosof B.M. (2020). Decreasing the Peril of Antimicrobial Resistance Through Enhanced Health Literacy in Outpatient Settings: An Underrecognized Approach to Advance Antimicrobial Stewardship. Adv. Ther..

[108] Osman O., Tanguichi H., Ikeda K., Park P., Tanabe-Hosoi S., Nagata S. (2010). Copper-resistant halophilic bacterium isolated from the polluted Maruit Lake, Egypt. J. Appl. Microbiol..

[109] Shinde V., Thombre R. (2016). Antibiotic resistance profiling of *Marine halophilic* bacteria and *Haloarchaea*. J. Appl. Pharm. Sci..

[110] Mukherjee A., Chettri B., Langpoklakpam J.S., Singh A.K., Chattopadhyay D. (2016). Draft Genome Sequence of Hydrocarbon-Degrading Enterobacter cloacae Strain S1:CND1, Isolated from Crude Oil-Contaminated Soil from the Noonmati Oil Refinery, Guwahati, Assam, India. Genome Announc..

[111] Mendis H.C., Thomas V.P., Schwientek P., Salamzade R., Chien J.-T., Waidyarathne P., Kloepper J., De La Fuente L. (2018). Strain-specific quantification of root colonization by plant growth promoting rhizobacteria *Bacillus firmus* I-1582 and *Bacillus amyloliquefaciens* QST713 in non-sterile soil and field conditions. PLoS ONE.

[112] Susič N., Žibrat U., Sinkovič L., Vončina A., Razinger J., Knapič M., Sedlar A., Širca S., Stare B.G. (2020). From Genome to Field—Observation of the Multimodal Nematicidal and Plant Growth-Promoting Effects of Bacillus firmus I-1582 on Tomatoes Using Hyperspectral Remote Sensing. Plants.

[113] Logan N. (2011). Bacillus and relatives in foodborne illness. J. Appl. Microbiol..

[114] Ehlers S., Merrill S. (2020). A Staphylococcus saprophyticus.

[115] Vaidya V.K. (2011). Horizontal Transfer of Antimicrobial Resistance by Extended-Spectrum β Lactamase-Producing Enterobacteriaceae. J. Lab. Physicians.

[116] Courvalin P. (1994). Transfer of antibiotic resistance genes between gram-positive and gram-negative bacteria. Antimicrob. Agents Chemother..

[117] Wang Y., Lu J., Mao L., Li J., Yuan Z., Bond P., Guo J. (2019). Antiepileptic drug carbamazepine promotes horizontal transfer of plasmid-borne multi-antibiotic resistance genes within and across bacterial genera. ISME J..

[118] Furlan J.P.R., dos Santos L.D.R., Moretto-Altarugio J., Ramos M.S., Gallo I.F.L., Alves G.D.A.D., Paulelli A.C., Rocha C.C.D.S., Cesila C.A., Gallimberti M. (2020). Occurrence and abundance of clinically relevant antimicrobial resistance genes in environmental samples after the Brumadinho dam disaster, Brazil. Sci. Total Environ..

[119] preprocessCore: A Collection of Pre-Processing Functions. R Package Version 1.54.0. https://github.com/bmbolstad/preprocessCore.

[120] McMurdie P.J., Holmes S. (2013). Phyloseq: An R Package for Reproducible Interactive Analysis and Graphics of Microbiome Census Data. PLoS ONE.

[121] R Foundation for Statistical Computing, Vienna, Austria. https://www.Rproject.org/.

[122] Yoon S.-H., Ha S.-M., Kwon S., Lim J., Kim Y., Seo H., Chun J. (2017). Introducing EzBioCloud: A taxonomically united database of 16S rRNA gene sequences and whole-genome assemblies. Int. J. Syst. Evol. Microbiol..

[123] Han R., Zhang X., Liu J., Long Q., Chen L., Liu D., Zhu D. (2017). Microbial community structure and diversity within hypersaline Keke Salt Lake environments. Can. J. Microbiol..

[124] Oueriaghli N., Castro D.J., Llamas I., Béjar V., Martínez-Checa F. (2018). Study of Bacterial Community Composition and Correlation of Environmental Variables in Rambla Salada, a Hypersaline Environment in South-Eastern Spain. Front. Microbiol..

[125] Bora N., Dodd C., Desmasures N. (2015). Diversity, Dynamics and Functional Role of Actinomycetes on European Smear Ripened Cheeses.

[126] Kumar S., Stecher G., Tamura K. (2016). MEGA7: Molecular Evolutionary Genetics Analysis Version 7.0 for Bigger Datasets Brief Communication. Mol. Biol. Evol..

[127] Catalase Test Protocol. https://asm.org/getattachment/72a871fc-ba92-4128-a194-6f1bab5c3ab7/Catalase-Test-Protocol.pdf.

[128] Oxidase Test Protocol. https://asm.org/getattachment/00ce8639-8e76-4acb-8591-0f7b22a347c6/oxidase-test-protocol-3229.pdf.

[129] Phillips K., Zaidan F., Elizondo O.R., Lowe K.L. (2012). Phenotypic characterization and 16S rDNA identification of culturable non-obligate halophilic bacterial communities from a hypersaline lake, La Sal del Rey, in extreme South Texas (USA). Aquat. Biosyst..

[130] Gonzalez J.M., Sáiz-Jiménez C. (2002). A fluorimetric method for the estimation of G+C mol% content in microorganisms by thermal denaturation temperature. Environ. Microbiol..

[131] Hammer Ø., Harper D.A.T., Ryan P.D. (2001). PAST: Paleontological statistics software package for education and data analysis. Palaeontol. Electron..

[132] Metsalu T., Vilo J. (2015). ClustVis: A web tool for visualizing clustering of multivariate data using Principal Component Analysis and heatmap. Nucleic Acids Res..

