# Supplementary material for: Ubiquitousness of Haloferax and Carotenoid Producing Genes in Arabian Sea Coastal Biosystems of India
Source: Mar Drugs. 2021 Jul 31;19(8):442. doi: 10.3390/md19080442 (PMC8400781; doi:10.3390/md19080442)
Supplement: Supplementary file 1 [file marinedrugs-19-00442-s001.zip › marinedrugs-1300741-SI.pdf]

Supplementary Information

Ubiquitousness of *Haloferax* and Carotenoid Producing Genes in Arabian Sea Coastal Biosystems of India

Jamseel Moopantakath[1], Madangchanok Imchen[1], Ranjith Kumavath[*1] and Rosa María Martínez-Espinosa[2,3]

1Department of Genomic Science, School of Biological Sciences, Central University of Kerala, Tejaswini Hills, Periya (PO) Kasaragod, Kerala 671320, India.

2Biochemistry and Molecular Biology Division, Agrochemistry and Biochemistry Department, Faculty of Sciences, University of Alicante, Ap. 99, E-03080 Alicante, Spain

3Multidisciplinary Institute for Environmental Studies "Ramón Margalef" University of Alicante, Ap. 99, E-03080 Alicante, Spain

* Correspondence: Dr. Ranjith Kumavath, Ph.D., FRSB,
Email: RNKumavath@gmail.com; RNKumavath@cukerala.ac.in, Tel. No. +91-8547648620

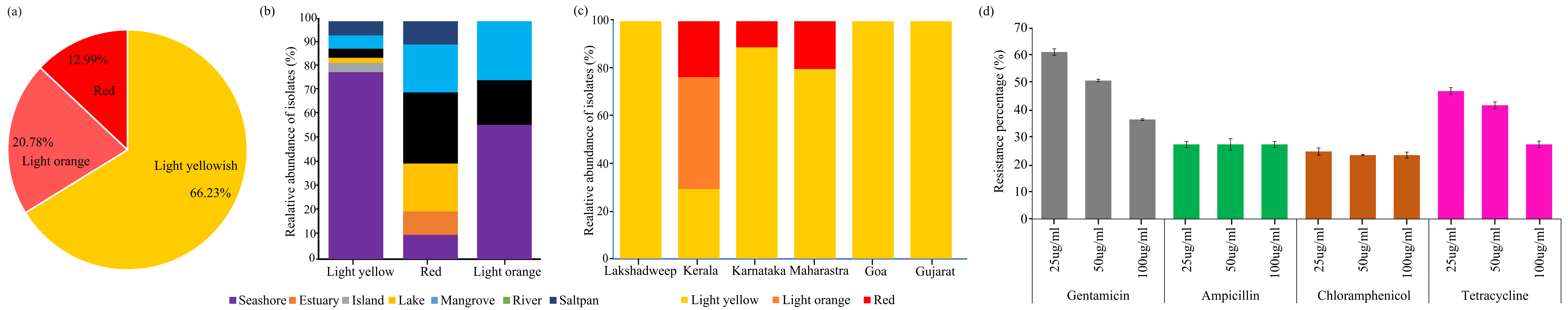

Figure S1: Pigmentation in the extreme halophiles grouped according to (a) pigmentation color, (b) biosystems, and (c) geography. (d) Antibiotic resistance profiles of the halotolerant isolates.

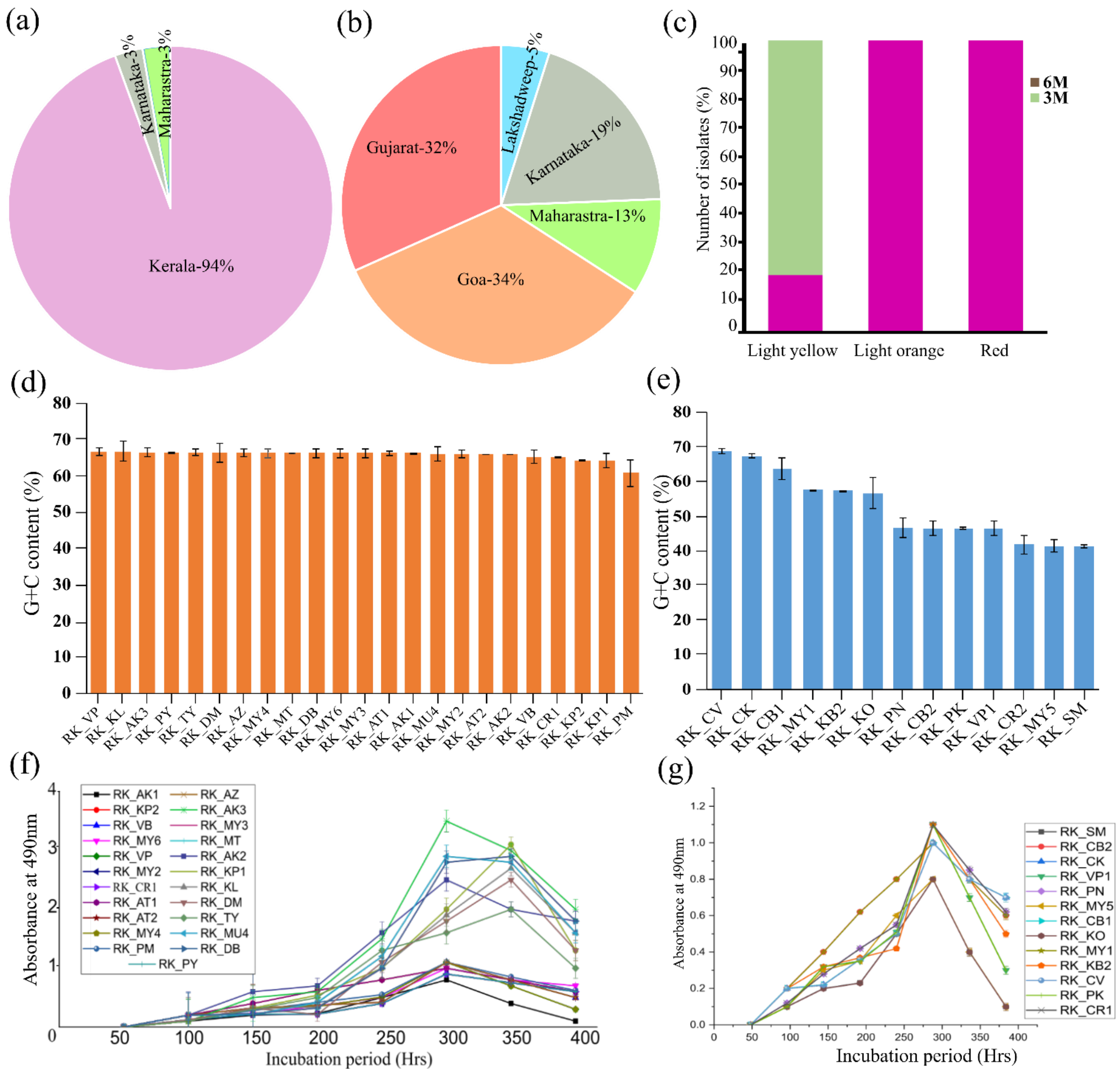

Figure S2: Distribution of halophilic isolates resistant to (a) 6M and (b) 3M NaCl enforced in MSG media. (c) 3M and 6M NaCl resistance isolated based on pigmentation. Yellow pigmented isolates were resistant only up to 3M NaCl. The G+C content of extreme halotolerant (d) archaea and (e) bacteria. Pigmentation production at various time points in 6M NaCl by halophilic (f) archaeal and (g) bacterial isolates.

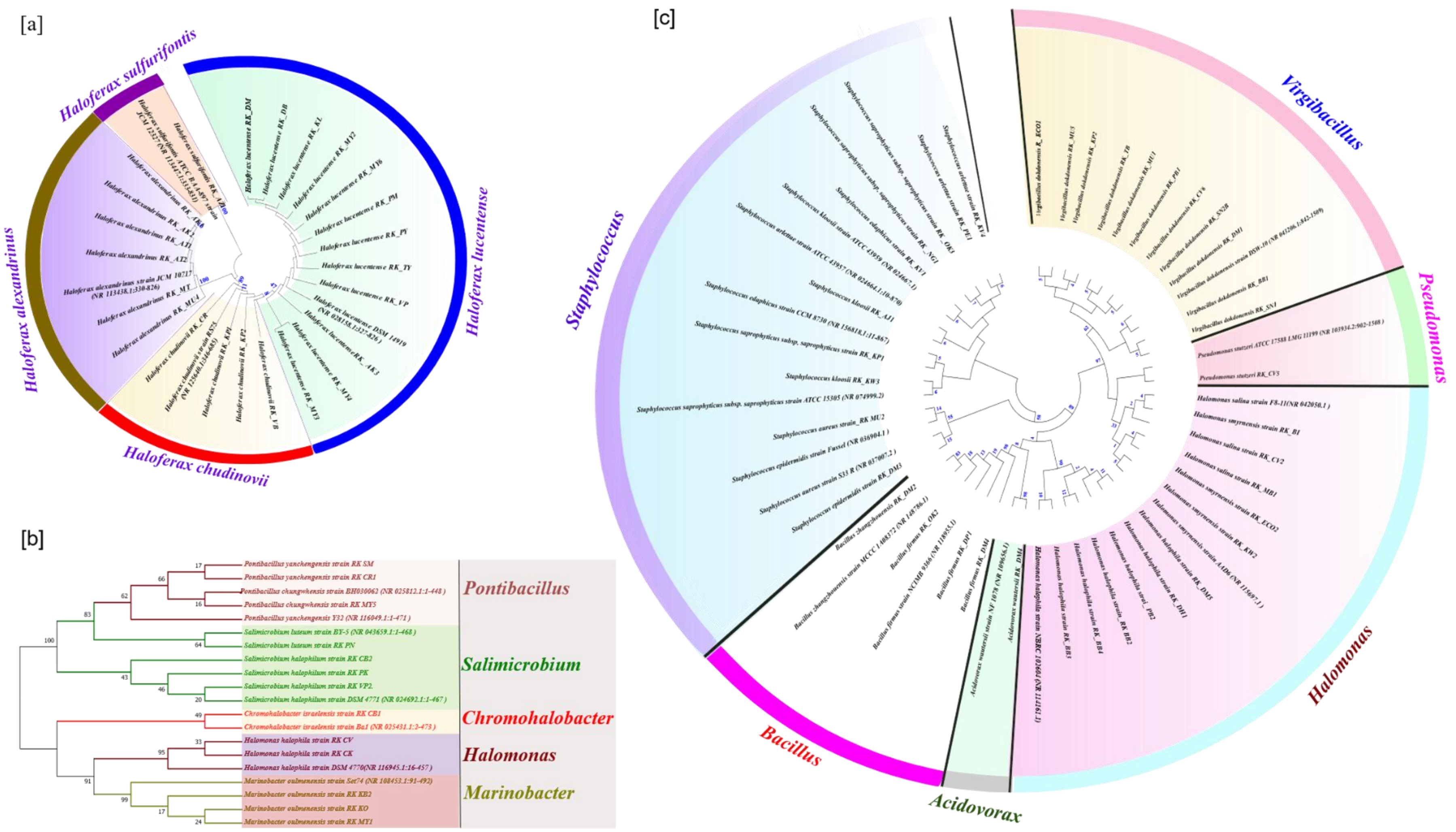

Figure S3: Phylogenetic tree, constructed using the neighbor-joining method, of halophilic (a) archaea and (b) bacteria isolated from Kerala coastal region, (c) bacterial halophiles from Gujarat, Maharashtra, Goa, Karnataka, and Lakshadweep coastal regions.

Table S1: Gram nature, motility, aerobic and pH parameters of the isolated halophiles.

S.No	Location	Name of the isolates	Gram nature	Motility	Anaerobic/Aerobic	Optimum pH
1	Kerala	RK_AK1	+Ve rods	Motile	Anaerobic & Aerobic	7-8
2	Kerala	RK_AK2	+Ve cocci	Motile	Anaerobic & Aerobic	7-8
3	Kerala	RK_AK3	+Ve cocci	Motile	Anaerobic & Aerobic	7-8
4	Kerala	RK_AT1	-Ve irregular	Motile	Anaerobic & Aerobic	7-8
5	Kerala	RK_AT2	+Ve rods	Motile	Anaerobic & Aerobic	7-8
6	Kerala	RK_AZ	+Ve coccobacilli	Motile	Anaerobic & Aerobic	7-8
7	Kerala	RK_CR	-Ve rods	Motile	Anaerobic & Aerobic	7-8
8	Kerala	RK_DM	-Ve cocci	Motile	Anaerobic & Aerobic	7-8
9	Kerala	RK_KL	+Ve cocci	Motile	Anaerobic & Aerobic	7-8
10	Kerala	RK_KP1	+Ve cocci	Motile	Anaerobic & Aerobic	7-8
11	Kerala	RK_KP2	+Ve rods	Motile	Anaerobic & Aerobic	7-8
12	Kerala	RK_MT	+Ve cocci	Motile	Anaerobic & Aerobic	7-8
13	Kerala	RK_MY2	-Ve rods	Motile	Anaerobic & Aerobic	7-8
14	Kerala	RK_MY3	+Ve cocci	Motile	Anaerobic & Aerobic	7-8
15	Kerala	RK_MY4	-Ve rods	Motile	Anaerobic & Aerobic	7-8
16	Kerala	RK_MY6	+Ve rods	Motile	Anaerobic & Aerobic	7-8
17	Kerala	RK_PM	+Ve rods	Motile	Anaerobic & Aerobic	7-8
18	Kerala	RK_PY	+Ve rods	Motile	Anaerobic & Aerobic	7-8
19	Kerala	RK_TY	+Ve cocci	Motile	Anaerobic & Aerobic	7-8
20	Kerala	RK_VB	-Ve rods	Motile	Anaerobic & Aerobic	7-8
21	Kerala	RK_VP	+Ve cocci	Motile	Anaerobic & Aerobic	7-8
22	Kerala	RK_CB1	-Ve rod	Motile	Anaerobic & Aerobic	7-8
23	Kerala	RK_CB2	+Ve cocci	Non Motile	Anaerobic & Aerobic	7-8
24	Kerala	RK_CK	-Ve rod	Motile	Anaerobic & Aerobic	7-8
25	Kerala	RK_CR1	+Ve rod	Motile	Anaerobic & Aerobic	7-8
26	Kerala	RK_CV	-Ve rod	Motile	Anaerobic & Aerobic	7-8
27	Kerala	RK_KB2	-Ve rod	Motile	Anaerobic & Aerobic	7-8
28	Kerala	RK_KO	-Ve rod	Motile	Anaerobic & Aerobic	7-8
29	Kerala	RK_MY1	-Ve rod	Motile	Anaerobic & Aerobic	7-8
30	Kerala	RK_MY5	+Ve rod	Non Motile	Anaerobic & Aerobic	7-8
31	Kerala	RK_PK	+Ve cocci	Non Motile	Anaerobic & Aerobic	7-8
32	Kerala	RK_PN	+Ve cocci	Non Motile	Anaerobic & Aerobic	7-8
33	Kerala	RK_SM	+Ve rod	Motile	Anaerobic & Aerobic	7-8
34	Kerala	RK_VP1	+Ve cocci	Non Motile	Anaerobic & Aerobic	7-8
35	Gujarat	RK_DM1	+Ve rod	Motile	Aerobic	7-8
36	Gujarat	RK_DM2	+Ve rod	Motile	Aerobic	7-8
37	Gujarat	RK_DM3	+Ve cocci	Non Motile	Aerobic	7-8
38	Gujarat	RK_DM4	-Ve rod	Motile	Aerobic	7-8
39	Gujarat	RK_DM5	-Ve rod	Motile	Aerobic	7-8
40	Gujarat	RK_NG1	+Ve cocci	Non Motile	Aerobic	7-8
41	Gujarat	RK_OK1	+Ve rod	Motile	Aerobic	7-8
42	Gujarat	RK_OK2	+Ve rod	Motile	Aerobic	7-8
43	Gujarat	RK_OK3	+Ve cocci	Non Motile	Aerobic	7-8
44	Gujarat	RK_PB1	+Ve rod	Motile	Aerobic	7-8
45	Gujarat	RK_PB2	-Ve rod	Motile	Aerobic	7-8
46	Gujarat	RK_SN1	+Ve rod	Motile	Aerobic	7-8
47	Gujarat	RK_SN2	+Ve rod	Motile	Aerobic	7-8
48	Goa	RK_AJ1	+Ve cocci	Non Motile	Aerobic	7-8
49	Goa	RK_BB1	+Ve rod	Motile	Aerobic	7-8
50	Goa	RK_BB2	-Ve rod	Motile	Aerobic	7-8
51	Goa	RK_BB3	-Ve rod	Motile	Aerobic	7-8
52	Goa	RK_BB4	-Ve rod	Motile	Aerobic	7-8
53	Goa	RK_CV1	+Ve cocci	Non Motile	Aerobic	7-8
54	Goa	RK_CV2	-Ve rod	Motile	Aerobic	7-8
55	Goa	RK_CV3	+Ve rod	Motile	Aerobic	7-8
56	Goa	RK_CV4	+Ve cocci	Non Motile	Aerobic	7-8
57	Goa	RK_CV5	-Ve rod	Motile	Aerobic	7-8
58	Goa	RK_CV6	+Ve rod	Motile	Aerobic	7-8
59	Goa	RK_DH1	-Ve rod	Motile	Aerobic	7-8
60	Goa	RK_MB1	-Ve rod	Motile	Aerobic	7-8
61	Goa	RK_PE1	+Ve cocci	Non Motile	Aerobic	7-8
62	Maharashtra	RK_DP1	+Ve rod	Motile	Aerobic	7-8
63	Maharashtra	RK_MU1	+Ve rod	Motile	Aerobic	7-8
64	Maharashtra	RK_MU2	+Ve cocci	Non Motile	Aerobic	7-8
65	Maharashtra	RK_MU4	+Ve cocci	Motile	Anaerobic & Aerobic	7-8
66	Maharashtra	RK_MU5	+Ve rod	Motile	Aerobic	7-8
67	Karnataka	RK_DB	+Ve cocci	Non Motile	Anaerobic & Aerobic	7-8
68	Karnataka	RK_ECO1	+Ve rod	Motile	Aerobic	7-8
69	Karnataka	RK_ECO2	-Ve rod	Motile	Aerobic	7-8
70	Karnataka	RK_KU1	+Ve cocci	Non Motile	Aerobic	7-8
71	Karnataka	RK_KU2	+Ve rod	Motile	Aerobic	7-8
72	Karnataka	RK_KW1	-Ve rod	Motile	Aerobic	7-8
73	Karnataka	RK_KW2	-Ve rod	Motile	Aerobic	7-8
74	Karnataka	RK_KW3	+Ve cocci	Non Motile	Aerobic	7-8
75	Karnataka	RK_TB	+Ve rod	Motile	Aerobic	7-8
76	Lakshadweep	RK_AI	+Ve cocci	Non Motile	Aerobic	7-8
77	Lakshadweep	RK_BI	-Ve rod	Motile	Aerobic	7-8

Table S2: Biochemical analysis of the microbial isolates: Catalase, Oxidases, urease (URE), , gelatinase (GEL), glucose (GLU), arabinose (ARA), amygdalin (AMY), xylose (XYL), esculin (ESC), saccharose (SAC), melibiose (MEL), lactose (LAC), mannose (MNE), β-galactosidase (ONPG), arginine dihydrolase (ADH), lysine decarboxylase (LDC), ornithine decarboxylase (ODC), citrate utilization (CIT), H2S production (H2S), tryptophane deaminase (TDA), indole production (IND), Voges–Proskauer (VP) mannitol (MAN), inositol (INO), sorbitol (SOR), rhamnose (RHAsalicin (SAL), glycerol (GLY), cellobiose (CEL), melezitose (MLZ), raffinose (RAF), and trehalose (TRE).

Characteristic	Catalase	Oxidases	URE	GEL	GLU	ARA	AMY	XYL	ESC	SAC	MEL	LAC	MNE	ONPG	ADH	LDC	ODC	CIT	H2S	TDA	IND	VP	MAN	INO	SOR	RHA	SAL	GLY	CEL	MLZ	RAF	TRE
RK_AK2	-	+	+	+	+	+	+	-	-	+	+	-	-	-	+	+	+	-	-	-	-	-	+	+	+	+	-	-	-	-	-	-
RK_AZ	-	-	+	+	+	+	+	-	-	+	+	-	-	-	+	-	+	-	-	-	-	-	-	+	+	+	-	-	-	-	-	-
RK_CR	-	+	-	-	+	+	+	-	-	+	+	-	-	+	+	-	-	-	+	-	-	-	+	-	-	+	-	-	-	-	-	-
RK_KB2	+	+	+	-	+	+	-	+	+	+	-	+	+	-	-	-	-	-	-	-	-	-	-	-	-	-	-	-	-	+	-	-
RK_MT	-	+	+	+	+	+	+	-	-	+	+	-	-	-	-	-	-	-	-	-	-	-	+	+	-	-	-	-	-	-	-	-
RK_AK3	-	+	+	+	+	+	-	+	+	-	-	+	+	-	-	-	-	-	-	-	-	-	-	-	-	-	-	-	-	-	-	-
RK_AT2	+	-	+	-	+	+	-	+	+	-	-	+	+	-	-	-	-	-	-	-	-	-	-	-	-	-	-	-	+	-	-	-
RK_PE1	+	-	+	-	+	-	-	-	+	-	-	+	-	-	-	-	-	+	-	-	-	+	+	-	+	-	-	-	-	-	-	-
RK_MY3	-	+	+	-	+	+	-	+	+	-	-	-	+	-	-	-	-	-	-	-	-	-	-	-	-	-	-	-	-	-	-	+
RK_ECO2	+	-	+	-	+	-	-	-	-	-	-	+	-	-	-	-	-	+	+	-	-	-	+	-	+	-	-	-	-	-	-	-
RK_MY2	+	-	-	+	-	+	-	+	-	-	-	+	+	-	-	-	-	-	-	-	-	-	-	-	-	-	-	-	-	-	+	-
RK_PY	-	+	+	-	+	-	+	-	-	-	-	-	-	-	+	-	-	-	-	-	-	+	+	-	-	-	-	-	-	-	-	-
RK_CV1	+	-	+	-	+	-	-	-	-	-	-	+	-	-	-	-	-	+	-	-	-	-	+	-	+	-	-	-	-	-	-	-
RK_KU1	+	-	+	-	+	-	-	-	-	-	-	+	-	-	-	-	-	-	+	-	-	+	-	-	+	-	-	-	-	-	-	-
RK_AI	+	+	+	-	+	-	-	-	-	-	-	+	-	-	-	-	-	-	-	-	-	-	+	-	+	-	-	-	-	-	-	-
RK_TY	-	+	+	+	-	+	-	-	-	-	+	-	-	-	-	-	-	-	-	-	-	-	+	-	-	-	-	-	-	-	-	-
RK_CR1	-	+	+	+	-	+	-	+	+	-	-	-	-	-	-	-	-	-	-	-	-	-	-	-	-	-	-	-	-	-	-	-
RK_AJ1	-	-	+	-	+	-	-	-	-	-	-	+	-	-	-	-	-	+	-	-	-	-	+	-	+	-	-	-	-	-	-	-
RK_MB1	+	+	+	-	+	-	-	-	-	-	-	+	-	-	-	-	-	-	-	-	-	-	-	-	+	-	-	-	-	-	-	-
RK_MU2	-	+	+	-	+	-	-	-	-	-	-	+	-	-	-	-	-	-	-	-	-	-	+	-	+	-	-	-	-	-	-	-
RK_KW3	-	+	+	-	+	-	-	-	-	-	-	+	-	-	-	-	-	+	-	-	-	-	-	-	+	-	-	-	-	-	-	-
RK_CK	-	+	+	-	-	-	-	+	+	-	-	+	-	-	-	-	-	-	-	-	-	-	-	-	-	-	-	-	-	-	-	-
RK_NG1	+	-	+	-	-	-	-	-	-	-	-	-	-	-	-	-	-	-	+	-	-	+	+	-	-	-	-	-	-	-	-	-
RK_OK1	+	+	+	-	-	-	-	-	-	-	-	-	-	-	-	-	-	+	-	-	-	-	+	-	-	-	-	-	-	-	-	-
RK_BB1	+	+	+	-	-	-	-	-	+	-	-	-	-	-	-	-	-	-	-	-	-	-	+	-	-	-	-	-	-	-	-	-
RK_BB4	+	+	+	-	+	-	-	-	-	-	-	-	-	-	-	-	-	-	-	-	-	-	+	-	-	-	-	-	-	-	-	-
RK_DP1	-	-	+	-	+	-	-	-	-	-	-	+	-	-	-	-	-	-	-	-	-	-	+	-	+	-	-	-	-	-	-	-
RK_ECO1	-	-	-	-	+	-	-	-	-	-	-	+	-	-	-	-	-	-	+	-	-	+	-	-	+	-	-	-	-	-	-	-
RK_KW1	-	-	+	-	+	-	-	-	-	-	-	+	-	-	-	-	-	-	-	-	-	-	+	-	+	-	-	-	-	-	-	-
RK_TB	-	-	-	-	+	-	-	-	+	-	-	+	-	-	-	-	-	-	+	-	-	-	-	-	+	-	-	-	-	-	-	-
RK_BI	+	-	+	-	+	-	-	-	-	-	-	+	-	-	-	-	-	-	-	-	-	-	-	-	+	-	-	-	-	-	-	-
RK_AK1	+	-	-	+	-	-	-	-	-	-	-	-	-	-	-	-	-	-	-	-	-	-	-	-	-	-	-	-	-	-	+	+
RK_DM	-	+	-	+	-	-	-	-	-	-	+	-	-	-	-	-	-	-	-	-	-	+	-	-	-	-	-	-	-	-	-	-
RK_MY1	+	+	+	-	-	-	-	-	+	-	-	-	-	-	-	-	-	-	-	-	-	-	-	-	-	-	-	-	-	-	-	-
RK_PK	+	-	+	-	+	-	-	-	-	-	-	-	-	-	+	-	-	-	-	-	-	-	-	-	-	-	-	-	-	-	-	-
RK_VP	+	-	+	-	+	-	-	-	-	-	-	-	-	-	+	-	-	-	-	-	-	-	-	-	-	-	-	-	-	-	-	-
RK_CV3	-	+	+	-	+	-	-	-	-	-	-	-	-	-	-	-	-	-	-	-	-	-	+	-	-	-	-	-	-	-	-	-
RK_CV6	-	+	+	-	-	-	-	-	-	-	-	-	-	-	-	-	-	+	-	-	-	-	+	-	-	-	-	-	-	-	-	-
RK_MU1	-	-	+	-	+	-	-	-	-	-	-	+	-	-	-	-	-	-	-	-	-	-	-	-	+	-	-	-	-	-	-	-
RK_MU4	-	+	-	-	+	-	-	-	-	-	-	+	-	-	-	-	-	-	-	-	-	-	-	-	+	-	-	-	-	-	-	-
RK_MU5	-	-	-	-	+	-	-	-	-	-	-	+	-	-	-	-	-	-	-	-	-	-	+	-	+	-	-	-	-	-	-	-
RK_KW2	-	-	-	-	+	-	-	-	+	-	-	+	-	-	-	-	-	-	-	-	-	-	-	-	+	-	-	-	-	-	-	-
RK_AT1	+	-	+	-	+	-	-	-	-	-	-	-	-	-	-	-	-	-	-	-	-	-	-	-	-	-	-	-	-	-	-	-
RK_KL	-	+	-	-	-	+	+	-	-	-	-	-	-	-	-	-	-	-	-	-	-	-	-	-	-	-	-	-	-	-	-	-
RK_KP2	-	+	+	+	-	-	-	-	-	-	-	-	-	-	-	-	-	-	-	-	-	-	-	-	-	-	-	-	-	-	-	-
RK_KP1	-	+	+	+	-	-	-	-	-	-	-	-	-	-	-	-	-	-	-	-	-	-	-	-	-	-	-	-	-	-	-	-
RK_MY4	-	-	+	+	+	-	-	-	-	-	-	-	-	-	-	-	-	-	-	-	-	-	-	-	-	-	-	-	-	-	-	-
RK_CV	-	+	+	+	-	-	-	-	-	-	-	-	-	-	-	-	-	-	-	-	-	-	-	-	-	-	-	-	-	-	-	-
RK_KO	+	+	+	-	-	-	-	-	-	-	-	-	-	-	-	-	-	-	-	-	-	-	-	-	-	-	-	-	-	-	-	-
RK_PN	+	+	-	+	-	-	-	-	-	-	-	-	-	-	-	-	-	-	-	-	-	-	-	-	-	-	-	-	-	-	-	-
RK_DM2	+	-	-	-	-	-	-	-	-	-	-	-	-	-	-	-	-	-	-	-	-	+	+	-	-	-	-	-	-	-	-	-
RK_DM3	+	-	-	-	-	-	-	-	+	-	-	-	-	-	-	-	-	+	-	-	-	-	-	-	-	-	-	-	-	-	-	-
RK_DM4	-	+	+	-	-	-	-	-	-	-	-	-	-	-	-	-	-	+	-	-	-	-	-	-	-	-	-	-	-	-	-	-
RK_OK2	+	-	+	-	-	-	-	-	-	-	-	-	-	-	-	-	-	-	-	-	-	-	+	-	-	-	-	-	-	-	-	-
RK_OK3	-	-	+	-	-	-	-	-	-	-	-	-	-	-	-	-	-	-	+	-	-	-	+	-	-	-	-	-	-	-	-	-
RK_PB1	-	-	-	-	-	-	-	-	-	-	-	-	-	-	-	-	-	-	+	-	-	+	+	-	-	-	-	-	-	-	-	-
RK_SN2	-	-	+	-	+	-	-	-	-	-	-	-	-	-	-	-	-	-	-	-	-	-	+	-	-	-	-	-	-	-	-	-
RK_CV4	-	+	+	-	-	-	-	-	-	-	-	-	-	-	-	-	-	-	+	-	-	-	-	-	-	-	-	-	-	-	-	-
RK_DH1	+	-	+	-	-	-	-	-	-	-	-	-	-	-	-	-	-	-	+	-	-	-	-	-	-	-	-	-	-	-	-	-
RK_DB	-	-	-	-	+	-	-	-	-	-	-	+	-	-	-	-	-	-	-	-	-	-	-	-	+	-	-	-	-	-	-	-
RK_KU2	-	-	-	-	+	-	-	-	-	-	-	+	-	-	-	-	-	-	-	-	-	-	-	-	+	-	-	-	-	-	-	-
RK_MY6	-	-	-	+	-	-	-	-	-	+	-	-	-	-	-	-	-	-	-	-	-	-	-	-	-	-	-	-	-	-	-	-
RK_PM	-	-	-	+	-	-	+	-	-	-	-	-	-	-	-	-	-	-	-	-	-	-	-	-	-	-	-	-	-	-	-	-
RK_VB	-	+	-	+	-	-	-	-	-	-	-	-	-	-	-	-	-	-	-	-	-	-	-	-	-	-	-	-	-	-	-	-
RK_VP1	-	-	-	-	-	-	+	-	-	-	-	-	-	+	-	-	-	-	-	-	-	-	-	-	-	-	-	-	-	-	-	-
RK_CB2	+	+	-	-	-	-	-	-	-	-	-	-	-	-	-	-	-	-	-	-	-	-	-	-	-	-	-	-	-	-	-	-
RK_MY5	+	-	+	-	-	-	-	-	-	-	-	-	-	-	-	-	-	-	-	-	-	-	-	-	-	-	-	-	-	-	-	-
RK_DM5	-	-	+	-	-	-	-	-	+	-	-	-	-	-	-	-	-	-	-	-	-	-	-	-	-	-	-	-	-	-	-	-
RK_PB2	-	-	+	-	-	-	-	-	-	-	-	-	-	-	-	-	-	-	-	-	-	-	+	-	-	-	-	-	-	-	-	-
RK_BB2	-	-	-	-	-	-	-	-	-	-	-	-	-	-	-	-	-	-	-	-	-	+	+	-	-	-	-	-	-	-	-	-
RK_BB3	-	-	+	-	-	-	-	-	-	-	-	-	-	-	-	-	-	-	+	-	-	-	-	-	-	-	-	-	-	-	-	-
RK_CV2	-	+	-	-	-	-	-	-	-	-	-	-	-	-	-	-	-	-	-	-	-	-	+	-	-	-	-	-	-	-	-	-
RK_CV5	-	+	+	-	-	-	-	-	-	-	-	-	-	-	-	-	-	-	-	-	-	-	-	-	-	-	-	-	-	-	-	-
RK_CB1	-	-	-	+	-	-	-	-	-	-	-	-	-	-	-	-	-	-	-	-	-	-	-	-	-	-	-	-	-	-	-	-
RK_SM	-	+	-	-	-	-	-	-	-	-	-	-	-	-	-	-	-	-	-	-	-	-	-	-	-	-	-	-	-	-	-	-
RK_DM1	-	-	-	-	-	-	-	-	+	-	-	-	-	-	-	-	-	-	-	-	-	-	-	-	-	-	-	-	-	-	-	-
RK_SN1	-	-	+	-	-	-	-	-	-	-	-	-	-	-	-	-	-	-	-	-	-	-	-	-	-	-	-	-	-	-	-	-

Table S3: 16s rRNA gene sequence similarity of halotolerant microbial isolates with location and accession number

Sequence ID	Location	Accession number	Closest taxon	16S rRNA gene similarity (%)
RK_AI	Agatti island, Lakshadweep	MT322528	*Staphylococcus arlettae strain ATCC 43957*	99.88
RK_AJ	Anjuna beach, Goa	MT322493	*Staphylococcus kloosii strain ATCC 43959*	99.74
RK_AT1	Anjuthengu beach, Thiruvananthapuram, Kerala	MT322460	*Haloferax alexandrinus strain JCM 10717*	99.80
RK_AT2	Anjuthengu beach, Thiruvananthapuram, Kerala	MT322461	*Haloferax alexandrinus strain JCM 10717*	100.00
RK_AK1	Asthamudi kayal, Kollam, Kerala	MT322457	*Haloferax alexandrinus strain JCM 10717*	100.00
RK_AK2	Asthamudi kayal, Kollam, Kerala	MT322458	*Haloferax alexandrinus strain JCM 10717*	99.80
RK_AK3	Asthamudi kayal, Kollam, Kerala	MT322459	*Haloferax lucentense strain Aa 2.2*	99.60
RK_AZ2	Azheekal beach, Alappuzha, Kerala	MT322462	*Haloferax sulfurifontis strain JCM 12327*	100.00
RK_BB1	Baga beach, Goa	MT322494	*Virgibacillus dokdonensis strain DSW10*	99.65
RK_BB2	Baga beach, Goa	MT322495	*Halomonas halophila strain NBRC 102604*	99.64
RK_BB3	Baga beach, Goa	MT322496	*Halomonas halophila strain NBRC 102604*	99.59
RK_BB4	Baga beach, Goa	MT322497	*Halomonas halophila strain NBRC 102604*	99.6
RK_BI	Bangaram island, Lakshadweep	MT322529	*Halomonas smyrnensis strain AAD6 16S*	100
RK_CK	Chavakkad, Thrissur, Kerala	MT322482	*Halomonas halophila strain DSM 4770*	100.00
RK_CV	Chavara beach, Kollam, Kerala	MT322484	*Halomonas halophila strain DSM 4770*	99.57
RK_CB1	Cheetuva beach, Thrissur, Kerala	MT322480	*Chromohalobacter israelensis strain Ba1*	99.79
RK_CB2	Cheetuva beach, Thrissur, Kerala	MT322481	*Salimicrobium halophilum strain DSM 4771*	99.57
RK_CR	Cheetuva river, Thrissur, Kerala	MT322483	*Haloferax chudinovii strain RS75*	100.00
RK_CR1	Cheetuva river, Thrissur,Kerala	MT322463	*Pontibacillus yanchengensis strain Y32*	99.15
RK_CV1	Colva beach, Goa	MT322498	*Staphylococcus edaphicus strain CCM 8730*	99.88
RK_CV2	Colva beach, Goa	MT322499	*Halomonas salina strain F811*	99.19
RK_CV3	Colva beach, Goa	MT322500	*Lysinibacillus cresolivorans strain SC03*	99.83
RK_CV4	Colva beach, Goa	MT322501	*Staphylococcus arlettae strain ATCC 43957*	99.88
RK_CV5	Colva beach, Goa	MT322502	*Pseudomonas stutzeri strain ATCC 17588*	99.84
RK_CV6	Colva beach, Goa	MT322503	*Virgibacillus dokdonensis strain DSW10 16S*	99.53
RK_DP1	Dana Pani beach, Maharashtra	MT322530	*Bacillus firmus strain NCIMB 9366*	100
RK_DB	Devbagh Beach, Karnataka	MT322464	*Haloferax lucentense strain JCM 9276*	99.76
RK_DM	Dharmadam, Kannur, Kerala	MT322465	*Haloferax lucentense strain JCM 9276*	100.00
RK_DH1	Dona Paula Beach, Goa	MT322504	*Halomonas halophila strain NBRC 102604*	100
RK_DM1	Dumas beach, Gujarat	MT322507	*Virgibacillus dokdonensis strain DSW10*	99.77
RK_DM2	Dumas beach, Gujarat	MT322508	*Bacillus zhangzhouensis strain MCCC 1A08372*	100
RK_DM3	Dumas beach, Gujarat	MT322509	*Staphylococcus epidermidis strain Fussel*	100
RK_DM4	Dumas beach, Gujarat	MT322510	*Bacillus firmus strain NCIMB 9366*	99
RK_DM5	Dumas beach, Gujarat	MT322511	*Halomonas halophila strain NBRC 102604*	100
RK_ECO1	Eco beach, Karnataka	MT322520	*Virgibacillus dokdonensis strain DSW10*	100
RK_ECO2	Eco beach, Karnataka	MT322521	*Halomonas smyrnensis strain AAD6*	100
RK_KL	Kallai, Kozhikode, Kerala	MT322466	*Haloferax lucentense strain JCM 9276*	100.00
RK_KW1	Karwar beach, Karnataka	MT322524	*Acidovorax wautersii strain NF 1078*	98.85
RK_KW2	Karwar beach, Karnataka	MT322525	*Halomonas smyrnensis strain AAD6 16S*	99.8
RK_KW3	Karwar beach, Karnataka	MT322526	*Staphylococcus kloosii strain ATCC 43959*	99.74
RK_KB2	Kollam beach, Kollam, Kerala	MT322485	*Marinobacter oulmenensis strain Set74*	99.26
RK_KO	Kovalam Beach, Thiruvananthapuram, Kerala	MT322486	*Marinobacter oulmenensis strain Set74*	100.00
RK_KU1	Kundapura beach, Karnataka	MT322522	*Staphylococcus saprophyticus subsp. saprophyticus strain ATCC 15305*	100
RK_KU2	Kundapura beach, Karnataka	MT322523	*Virgibacillus dokdonensis strain DSW10*	100
RK_KP1	Kureepuzha, Kollam, Kerala	MT322467	*Haloferax chudinovii strain RS75*	99.80
RK_KP2	Kureepuzha, Kollam, Kerala	MT322468	*Haloferax chudinovii strain RS75*	99.80
RK_MT	Mattancherry, Ernakulam, Kerala	MT322469	*Haloferax alexandrinus strain JCM 10717*	99.80
RK_MY1	Mayyanad, Kollam, Kerala	MT322487	*Marinobacter oulmenensis strain Set74*	98.48
RK_MY2	Mayyanad, Kollam, Kerala	MT322471	*Haloferax lucentense strain JCM 9276*	99.80
RK_MY3	Mayyanad, Kollam, Kerala	MT322472	*Haloferax lucentense strain Aa 2.2*	99.60
RK_MY4	Mayyanad, Kollam, Kerala	MT322473	*Haloferax lucentense strain Aa 2.2*	99.58
RK_MY5	Mayyanad, Kollam, Kerala	MT322488	*Pontibacillus chungwhensis strain BH030062*	99.55
RK_MY6	Mayyanad, Kollam, Kerala	MT322474	*Haloferax lucentense strain JCM 9276*	99.80
RK_MB1	Morbar beach, Goa	MT322505	*Halomonas salina strain F811 16S*	98.97
RK_MU1	Mulund saltpan, Maharashtra	MT322531	*Virgibacillus dokdonensis strain DSW10*	99.42
RK_MU2	Mulund saltpan, Maharashtra	MT322532	*Staphylococcus aureus strain S33 R*	99.48
RK_MU5	Mulund saltpan, Maharashtra	MT322533	*Virgibacillus dokdonensis strain DSW10*	99.55
RK_MU4	Mulund Saltpan, Maharashtra	MT322470	*Haloferax alexandrinus strain JCM 10717*	99.80
RK_NG1	Nargoal beach, Gujarat	MT322512	*Staphylococcus sp. clone WLA214 16S ribosomal RNA gene,*	100
RK_OK1	Okha beach, Gujarat	MT322513	*Virgibacillus dokdonensis strain DSW10*	99.55
RK_OK2	Okha beach, Gujarat	MT322514	*Bacillus firmus strain NBRC 15306*	99.49
RK_OK3	Okha beach, Gujarat	MT322515	*Staphylococcus saprophyticus subsp. saprophyticus strain ATCC 15305*	100
RK_PK	Paddnakadappuram, Kasaragod, Kerala	MT322489	*Salimicrobium halophilum strain NBRC 102426*	99.65
RK_PE1	Paloelum beach, Goa	MT322506	*Staphylococcus arlettae strain ATCC 43957*	99.88
RK_PM	Payyambalam beach, Kannur, Kerala	MT322475	*Haloferax lucentense strain JCM 9276*	99.68
RK_PY	Payyanur, Kannur, Kerala	MT322476	*Haloferax lucentense strain JCM 9276*	100.00
RK_PN	Ponnani, Malappuram, Kerala	MT322490	*Salimicrobium luteum strain BY-5*	99.79
RK_PB1	Porbandar beach, Gujarat	MT322516	*Virgibacillus dokdonensis strain DSW10*	99.55
RK_PB2	Porbandar beach, Gujarat	MT322517	*Halomonas halophila strain NBRC 102604*	99.27
RK_SM	Shamugam beach, Thiruvananthapuram, Kerala	MT322491	*Pontibacillus yanchengensis strain Y32*	99
RK_SN1	Somnath beach, Gujarat	MT322518	*Virgibacillus dokdonensis strain DSW10*	99.88
RK_SN2	Somnath beach, Gujarat	MT322519	*Virgibacillus dokdonensis strain DSW10*	100
RK_TB	Tannirbhavi beach, Karnataka	MT322527	*Virgibacillus dokdonensis strain DSW10*	100
RK_TY	Thalassery, Kannur, Kerala	MT322477	*Haloferax lucentense strain JCM 9276*	99.79
RK_VP	Valapattanam, Kannur, Kerala	MT322479	*Haloferax lucentense strain JCM 9276*	100.00
RK_VP1	Valiya paramba,Kasargod,Kerala	MT322492	*Salimicrobium halophilum strain NBRC 102426*	100.00
RK_VB	Varkala beach, Thiruvananthapuram, Kerala	MT322478	*Haloferax chudinovii strain RS75*	99.79

Table S4: **The metagenome data set were retrieved from the MG-RAST server for the comparative analysis of the open sea and coastal microbiome. Sampling with a distance of 100+ km away, calculated manually with https://www.freemaptools.com/measure-distance.htm, from coastline was considered as open sea.**

S.No	MG-RAST_id	Biosystem	Km	Latitude	Longitude	Country	Location
1	mgm4441121.3	Open Ocean	7702.546	-15.2778	-148.224	French Polynesia	Polynesia Archipelagos - Tikehau Lagoon
2	mgm4441124.3	Open Ocean	1977.819	-2.58194	-97.8514		Tropical South Pacific
3	mgm4441133.3	Open Ocean	1176.334	-12.0925	96.88167	Australia	Indian Ocean - Coccos Keeling_ Inside Lagoon
4	mgm4441134.3	Open Ocean	1801.178	-10.4461	88.3027		Indian Ocean
5	mgm4441135.3	Open Ocean	335.7092	-26.035	50.123	Madagascar	Indian Ocean - Madagascar Waters
6	mgm4441139.3	Open Ocean	1122.212	-12.0925	96.88167	Australia	Indian Ocean - Coccos Keeling_ Inside Lagoon
7	mgm4441139.4	Open Ocean	813.1854	-30.8983	40.4203		Indian Ocean - International waters between Madagascar and South Africa
8	mgm4441146.3	Open Ocean	4878.034	-10.1314	-135.449	French Polynesia	Tropical South Pacific - 201 miles from F. Polynesia
9	mgm4441147.3	Open Ocean	2368.085	-8.505	80.3756		Indian Ocean
10	mgm4441148.3	Open Ocean	2891.074	-4.6136	55.5086	Seychelles	Indian Ocean - St. Anne Island_ Seychelles
11	mgm4441149.3	Open Ocean	1746.766	-4.635	56.8361	Seychelles	Outside Seychelles_ Indian Ocean
12	mgm4441150.3	Open Ocean	2270.253	-4.6625	60.5231		Indian Ocean
13	mgm4441151.3	Open Ocean	1705.052	-23.2161	52.3061		Indian Ocean - International Water Outside of Reunion Island
14	mgm4441155.3	Open Ocean	2699.835	-10.9436	92.05889		Indian Ocean
15	mgm4441156.3	Open Ocean	2114.485	-9.59694	84.1957		Indian Ocean
16	mgm4441572.3	Open Ocean	1153.562	31.175	-64.324	Bermuda	Sargasso Sea
17	mgm4441574.3	Open Ocean	1116.515	32.1748	-64.0102	Bermuda	Sargasso Sea
18	mgm4441575.3	Open Ocean	1285.029	31.175	-64.324	Bermuda	Sargasso Sea
19	mgm4441576.3	Open Ocean	1059.64	32.1667	-64.5	Bermuda	Sargasso Sea
20	mgm4441578.3	Coastal	21.37209	32.1667	-64.5	Bermuda	Sargasso Sea
21	mgm4441579.3	Open Ocean	1187.117	42.5031	-67.24	Canada	Gulf of Maine
22	mgm4441581.3	Coastal	0	44.69	-63.637	Canada	Bedford Basin_ Nova Scotia
23	mgm4441582.3	Coastal	0	44.112	-64.9467	Canada	Bay of Fundy_ Nova Scotia
24	mgm4441583.3	Coastal	0	41.4858	-71.3511	USA	Newport Harbor_ RI
25	mgm4441587.3	Open Ocean	158.9227	20.5225	-85.4136	Mexico	Yucatan Channel
26	mgm4441588.3	Open Ocean	241.2568	18.0367	-83.785	Honduras	Caribbean Sea_ Rosario Bank
27	mgm4441590.3	Coastal	0	9.1644	-79.8361	Panama	Lake Gatun_ Panama
28	mgm4441591.3	Coastal	40.29797	8.1292	-79.6911	Panama	Eastern Tropical Pacific_ Gulf of Panama
29	mgm4441592.3	Open Ocean	210.6631	6.4927	-82.9039	Panama	Eastern Tropical Pacific_ 250 miles from Panama City
30	mgm4441593.3	Open Ocean	542.2363	5.55278	-87.0878	Costa Rica	Eastern Tropical Pacific_ Dirty Rock_ Cocos Island
31	mgm4441595.3	Open Ocean	1173.131	1.2161	-90.4228	Ecuador	Galapagos Islands_ Devil
32	mgm4441603.3	Open Ocean	5960.672	-17.4758	-149.812	French Polynesia	Polynesia Archipelagos - Moorea_ Cooks Bay
33	mgm4441606.3	Open Ocean	220.0456	12.0925	96.88167	Australia	Indian Ocean - Coccos Keeling_ Inside Lagoon
34	mgm4441607.3	Open Ocean	2426.022	-10.4461	88.3027		Indian Ocean
35	mgm4441608.3	Open Ocean	2404.07	-10.4461	88.30278		Indian Ocean
36	mgm4441609.3	Open Ocean	2455.119	-8.505	80.3756		Indian Ocean
37	mgm4441610.3	Open Ocean	1716.06	-7.0075	76.3314		Indian Ocean
38	mgm4441611.3	Coastal	0	-4.99028	64.9767		Indian Ocean - 500 Miles west of the Seychelles in the Indian Ocean
39	mgm4441613.3	Open Ocean	1822.18	-4.6136	55.5086	Seychelles	Indian Ocean - St. Anne Island_ Seychelles
40	mgm4441614.3	Open Ocean	1057.677	-29.3489	43.2156		Indian Ocean - International water between Madagascar and South Africa
41	mgm4441615.3	Open Ocean	811.1416	-30.8983	40.4203		Indian Ocean - International waters between Madagascar and South Africa
42	mgm4441616.3	Open Ocean	587.2174	-32.3992	36.5919		Indian Ocean - International water between Madagascar and South Africa
43	mgm4441617.3	Coastal	24.59078	-6.3167	39.55	Tanzania	Indian Ocean - East coast Zanzibar (Tanzania)_ offshore Paje lagoon
44	mgm4441618.3	Coastal	0	-6.1167	39.1167	Tanzania	Indian Ocean - West coast Zanzibar (Tanzania)_ harbour region
45	mgm4441659.3	Coastal	45.51225	32.5069	-79.2639	USA	North American East Coast_ South of Charleston_ SC
46	mgm4441660.3	Open Ocean	373.1908	24.1747	-84.344	USA	Caribbean Sea_ Gulf of Mexico
47	mgm4441661.3	Coastal	0	5.64	-86.565	Costa Rica	Eastern Tropical Pacific_ 30 miles from Cocos Island
48	mgm4561566.3	Coastal	0	31.50805	35.471	Dead Sea	Israel
49	mgm4722281.3	Coastal	80.11314	42.2038	17.715	Mediterranean Sea	Mediterranean Sea
50	mgm4722290.3	Open Ocean	427.1843	-60.2287	-60.6476	Southern Ocean	Southern Ocean
51	mgm4722304.3	Coastal	0	18.3967	39.875	Indian Ocean	Red Sea
52	mgm4722291.3	Open Ocean	434.0562	20.8183	63.5047	Indian Ocean	Indian Ocean
53	mgm4722283.3	Coastal	67.12574	27.16	34.835	Indian Ocean	Red Sea
54	mgm4722302.3	Open Ocean	498.0598	-5.2529	-85.1545	Pacific Ocean	South Pacific Ocean
55	mgm4722300.3	Coastal	0	35.759	14.2574	Mediterranean Sea	Mediterranean Sea
56	mgm4722298.3	Coastal	41.27967	36.5533	-6.5669	Atlantic Ocean	North Atlantic Ocean
57	mgm4722307.3	Open Ocean	552.5361	-47.1863	-58.2902	Atlantic Ocean	South Atlantic Ocean
58	mgm4722277.3	Coastal	0	-20.9354	-35.1803	Atlantic Ocean	South Atlantic Ocean
59	mgm4722314.3	Open Ocean	552.6487	-47.1863	-58.2902	Atlantic Ocean	South Atlantic Ocean
60	mgm4722301.3	Open Ocean	5288.852	0.0003	-153.676	Pacific Ocean	South Pacific Ocean
61	mgm4722294.3	Open Ocean	106.0236	23.36	37.2183	Indian Ocean	Red Sea
62	mgm4722312.3	Coastal	96.75376	39.3888	19.3905	Mediterranean Sea	Mediterranean Sea
63	mgm4722303.3	Open Ocean	1128.762	0.0033	71.6428	Indian Ocean	Indian Ocean
64	mgm4722293.3	Coastal	94.99958	21.9467	38.2517	Indian Ocean	Red Sea
65	mgm4722287.3	Open Ocean	5288.498	-8.9111	-142.557	Pacific Ocean	South Pacific Ocean
66	mgm4722310.3	Coastal	96.93079	21.9467	38.2517	Indian Ocean	Red Sea
67	mgm4722276.3	Open Ocean	1670.821	-20.4091	-3.1759	Atlantic Ocean	South Atlantic Ocean
68	mgm4722285.3	Open Ocean	368.6041	25.5264	-88.394	Atlantic Ocean	North Atlantic Ocean
69	mgm4722282.3	Open Ocean	464.6015	6.0001	73.8955	Indian Ocean	Indian Ocean
70	mgm4722315.3	Open Ocean	3414.529	31.5213	-158.996	Pacific Ocean	North Pacific Ocean
71	mgm4779568.3	Coastal	0	-68.5602	78.19686	Antarctica	Deep_lake
72	mgm4779570.3	Coastal	0	37.78556	-0.78722	Spain	Mar_menor
73	mgm4779571.3	Coastal	0.257495	23.94056	70.18778	India	Kutch_Desert
74	mgm4779572.3	Coastal	0	51.62	79.84	Russia	Kulunda_steppe
75	mgm4779573.3	Coastal	0	-35.32	142.8	Australia	Lake_tyrrel
76	mgm4779575.3	Coastal	0	39.33167	-0.35222	Spain	Albufera
77	mgm4779576.3	Coastal	0	-35.32	142.8	Australia	Lake_tyrrel
78	mgm4779577.3	Coastal	0	-35.32	142.8	Australia	Lake_tyrrel
79	mgm4779578.3	Coastal	0	51.67	79.91	Russia	Kulunda_steppe
80	mgm4779580.3	Coastal	0	49.73046	-119.874	Canada	British_Columbia
81	mgm4779582.3	Coastal	0	38.2	-0.6	Spain	Santa_Pola
82	mgm4779583.3	Coastal	0	79.11667	-90.35	Canada	Lost_hammer
83	mgm4779585.3	Coastal	0	-33.4261	121.689	Australia	Yilgarn_Craton
84	mgm4779586.3	Coastal	0	37.78556	-0.78722	Spain	Mar_menor
85	mgm4779587.3	Coastal	0	-35.32	142.8	Australia	Lake_tyrrel
86	mgm4779588.3	Coastal	0	-35.32	142.8	Australia	Lake_tyrrel
87	mgm4779589.3	Coastal	0	49.73046	-119.874	Canada	British_Columbia
88	mgm4779590.3	Coastal	0	-35.32	142.8	Australia	Lake_tyrrel
89	mgm4779591.3	Coastal	0	23.94083	70.18833	India	Kutch_Desert

90	mgm4779592.3	Coastal	0	-35.32	142.8	Australia	Lake_tyrrel
91	mgm4779593.3	Coastal	0	23.94139	70.18833	India	Kutch_Desert
92	mgm4779594.3	Coastal	0	-35.32	142.8	Australia	Lake_tyrrel
93	mgm4779595.3	Coastal	0	51.73	79.87	Russia	Kulunda_steppe
94	mgm4779596.3	Coastal	0	51.65	79.75	Russia	Kulunda_steppe
95	mgm4779598.3	Coastal	0	38.2	-0.6	Spain	Santa_pola
96	mgm4779600.3	Coastal	0	37.78556	-0.78722	Spain	Mar_menor
97	mgm4779601.3	Coastal	0	38.2	-0.6	Spain	Santa_Pola
98	mgm4779602.3	Coastal	0	51.62	79.84	Russia	Kulunda_steppe
99	mgm4779603.3	Coastal	0	38.2	-0.6	Spain	Santa_Pola
100	mgm4779604.3	Coastal	0	23.7925	71.00556	India	Kutch_Desert
101	mgm4779605.3	Coastal	0	51.65	79.75	Russia	Kulunda_steppe
102	mgm4779606.3	Coastal	0	-68.5602	78.19686	Antarctica	Deep_lake
103	mgm4779607.3	Coastal	0	51.73	79.87	Russia	Kulunda_steppe
104	mgm4779608.3	Coastal	0	23.835	69.51889	India	Kutch_Desert
105	mgm4779609.3	Coastal	0	-35.32	142.8	Australia	Lake_tyrrel
106	mgm4779610.3	Coastal	0	-35.32	142.8	Australia	Lake_tyrrel
107	mgm4779611.3	Coastal	0	-33.4261	121.689	Australia	Yilgarn_Craton
108	mgm4779613.3	Coastal	0	49.73046	-119.874	Canada	British_Columbia
109	mgm4779616.3	Coastal	0	23.81083	78.98333	India	Kutch_Desert
110	mgm4779617.3	Coastal	0	-35.32	142.8	Australia	Lake_tyrrel
111	mgm4779618.3	Coastal	0	39.33167	-0.35222	Spain	Albufera
112	mgm4779619.3	Coastal	0.241402	37.21889	-7.33806	Spain	Isla_Cristina
113	mgm4581707.3	Coastal	59.83541	31.852	29.9822	Egypt	Egypt
114	mgm4581709.3	Coastal	59.83541	31.852	29.9822	Egypt	Egypt
115	mgm4581710.3	Coastal	59.83541	31.852	29.9822	Egypt	Egypt
116	mgm4581711.3	Coastal	59.83541	31.852	29.9822	Egypt	Egypt
117	mgm4581712.3	Coastal	59.83541	31.852	29.9822	Egypt	Egypt
118	mgm4581713.3	Coastal	59.83541	31.852	29.9822	Egypt	Egypt
119	mgm4849668.3	Coastal	44.53055	-31.9998	115.5145	Australia	Rottnest Island
120	mgm4849678.3	Coastal	44.53055	-31.9966	115.5315	Australia	Rottnest Island
121	mgm4849698.3	Coastal	44.53055	-31.9966	115.5315	Australia	Rottnest Island
122	mgm4849710.3	Coastal	44.53055	-31.9966	115.5315	Australia	Rottnest Island
123	mgm4849716.3	Coastal	41.77857	-31.9956	115.5369	Australia	Rottnest Island
124	mgm4849786.3	Coastal	41.77857	-32.0027	115.526	Australia	Rottnest Island
125	mgm4849790.3	Coastal	41.77857	-31.9974	115.5366	Australia	Rottnest Island
126	mgm4849796.3	Coastal	41.77857	-32.0027	115.526	Australia	Rottnest Island
127	mgm4670271.3	Coastal	80.30627	-63.18	-60.67	Antarctica	deception_island
128	mgm4670272.3	Open Ocean	159.5665	-62.96	-60.71	Antarctica	deception_island
129	mgm4670273.3	Open Ocean	159.5665	-62.96	-60.71	Antarctica	deception_island
130	mgm4670275.3	Coastal	80.30627	-63.18	-60.67	Antarctica	deception_island
131	mgm4670276.3	Open Ocean	159.5665	-62.96	-60.71	Antarctica	deception_island
132	mgm4670277.3	Coastal	80.30627	-63.18	-60.67	Antarctica	deception_island
133	mgm4670279.3	Coastal	80.30627	-63.18	-60.67	Antarctica	deception_island
134	mgm4670280.3	Open Ocean	159.5665	-62.96	-60.71	Antarctica	deception_island
135	mgm4670281.3	Coastal	80.30627	-63.18	-60.67	Antarctica	deception_island
136	mgm4670282.3	Open Ocean	159.5665	-62.96	-60.71	Antarctica	deception_island
137	mgm4670283.3	Open Ocean	159.5665	-62.96	-60.71	Antarctica	deception_island
138	mgm4670284.3	Open Ocean	159.5665	-62.96	-60.71	Antarctica	deception_island
139	mgm4670285.3	Open Ocean	159.5665	-62.96	-60.71	Antarctica	deception_island
140	mgm4670286.3	Coastal	80.30627	-63.18	-60.67	Antarctica	deception_island
141	mgm4670287.3	Coastal	80.30627	-63.18	-60.67	Antarctica	deception_island
142	mgm4670288.3	Coastal	80.30627	-63.18	-60.67	Antarctica	deception_island
143	mgm4581719.3	Coastal	95.6755	23.02528	60.86639	Pakistan	Indus River Basin
144	mgm4581720.3	Coastal	95.6755	23.02528	60.86639	Pakistan	Indus River Basin
145	mgm4581721.3	Coastal	95.6755	23.02528	60.86639	Pakistan	Indus River Basin
146	mgm4581722.3	Coastal	95.6755	23.02528	60.86639	Pakistan	Indus River Basin
147	mgm4581723.3	Coastal	95.6755	23.02528	60.86639	Pakistan	Indus River Basin
148	mgm4581724.3	Coastal	95.6755	23.02528	60.86639	Pakistan	Indus River Basin
149	mgm4581725.3	Coastal	95.6755	23.02528	60.86639	Pakistan	Indus River Basin
150	mgm4581726.3	Coastal	95.6755	23.02528	60.86639	Pakistan	Indus River Basin
151	mgm4581729.3	Coastal	95.6755	23.02528	60.86639	Pakistan	Indus River Basin
152	mgm4581730.3	Coastal	95.6755	23.02528	60.86639	Pakistan	Indus River Basin
153	mgm4581731.3	Coastal	95.6755	23.02528	60.86639	Pakistan	Indus River Basin
154	mgm4581732.3	Coastal	95.6755	23.02528	60.86639	Pakistan	Indus River Basin
155	mgm4581733.3	Coastal	95.6755	23.02528	60.86639	Pakistan	Indus River Basin
156	mgm4581734.3	Coastal	95.6755	23.02528	60.86639	Pakistan	Indus River Basin
157	mgm4581735.3	Coastal	95.6755	23.02528	60.86639	Pakistan	Indus River Basin
158	mgm4581736.3	Coastal	95.6755	23.02528	60.86639	Pakistan	Indus River Basin
159	mgm4581737.3	Coastal	95.6755	23.02528	60.86639	Pakistan	Indus River Basin
160	mgm4581738.3	Coastal	95.6755	23.02528	60.86639	Pakistan	Indus River Basin
161	mgm4581739.3	Coastal	95.6755	23.02528	60.86639	Pakistan	Indus River Basin
162	mgm4581740.3	Coastal	95.6755	23.02528	60.86639	Pakistan	Indus River Basin
163	mgm4581741.3	Coastal	95.6755	23.02528	60.86639	Pakistan	Indus River Basin
164	mgm4581742.3	Coastal	95.6755	23.02528	60.86639	Pakistan	Indus River Basin
165	mgm4581745.3	Coastal	95.6755	23.02528	60.86639	Pakistan	Indus River Basin
166	mgm4581746.3	Coastal	95.6755	23.02528	60.86639	Pakistan	Indus River Basin
167	mgm4581749.3	Coastal	95.6755	23.02528	60.86639	Pakistan	Indus River Basin
168	mgm4581751.3	Coastal	95.6755	23.02528	60.86639	Pakistan	Indus River Basin
169	mgm4581753.3	Coastal	95.6755	23.02528	60.86639	Pakistan	Indus River Basin
170	mgm4581754.3	Coastal	95.6755	23.02528	60.86639	Pakistan	Indus River Basin
171	mgm4581755.3	Coastal	95.6755	23.02528	60.86639	Pakistan	Indus River Basin
172	mgm4581758.3	Coastal	95.6755	23.02528	60.86639	Pakistan	Indus River Basin
173	mgm4581759.3	Coastal	95.6755	23.02528	60.86639	Pakistan	Indus River Basin
174	mgm4581760.3	Coastal	95.6755	23.02528	60.86639	Pakistan	Indus River Basin
175	mgm4581761.3	Coastal	95.6755	23.02528	60.86639	Pakistan	Indus River Basin
176	mgm4581762.3	Coastal	95.6755	23.02528	60.86639	Pakistan	Indus River Basin
177	mgm4581763.3	Coastal	95.6755	23.02528	60.86639	Pakistan	Indus River Basin
178	mgm4581764.3	Coastal	95.6755	23.02528	60.86639	Pakistan	Indus River Basin
179	mgm4581765.3	Coastal	95.6755	23.02528	60.86639	Pakistan	Indus River Basin
180	mgm4581766.3	Coastal	95.6755	23.02528	60.86639	Pakistan	Indus River Basin
181	mgm4581767.3	Coastal	95.6755	23.02528	60.86639	Pakistan	Indus River Basin
182	mgm4581769.3	Coastal	95.6755	23.02528	60.86639	Pakistan	Indus River Basin
183	mgm4581770.3	Coastal	95.6755	23.02528	60.86639	Pakistan	Indus River Basin
184	mgm4581771.3	Coastal	95.6755	23.02528	60.86639	Pakistan	Indus River Basin
185	mgm4581772.3	Coastal	95.6755	23.02528	60.86639	Pakistan	Indus River Basin

186	mgm4581773.3	Coastal	95.6755	23.02528	60.86639	Pakistan	Indus River Basin
187	mgm4581776.3	Coastal	95.6755	23.02528	60.86639	Pakistan	Indus River Basin
188	mgm4581778.3	Coastal	95.6755	23.02528	60.86639	Pakistan	Indus River Basin
189	mgm4581779.3	Coastal	95.6755	23.02528	60.86639	Pakistan	Indus River Basin
190	mgm4581780.3	Coastal	95.6755	23.02528	60.86639	Pakistan	Indus River Basin
191	mgm4581782.3	Coastal	95.6755	23.02528	60.86639	Pakistan	Indus River Basin
192	mgm4581783.3	Coastal	95.6755	23.02528	60.86639	Pakistan	Indus River Basin
193	mgm4581784.3	Coastal	95.6755	23.02528	60.86639	Pakistan	Indus River Basin
194	mgm4581785.3	Coastal	95.6755	23.02528	60.86639	Pakistan	Indus River Basin
195	mgm4581787.3	Coastal	95.6755	23.02528	60.86639	Pakistan	Indus River Basin
196	mgm4581788.3	Coastal	95.6755	23.02528	60.86639	Pakistan	Indus River Basin
197	mgm4581789.3	Coastal	95.6755	23.02528	60.86639	Pakistan	Indus River Basin
198	mgm4581790.3	Coastal	95.6755	23.02528	60.86639	Pakistan	Indus River Basin
199	mgm4671023.3	Coastal	95.6755	23.02528	60.86639	Pakistan	Arabian Sea
200	mgm4671024.3	Coastal	95.6755	23.02528	60.86639	Pakistan	Arabian Sea
201	mgm4671025.3	Coastal	95.6755	23.02528	60.86639	Pakistan	Arabian Sea
202	mgm4667575.3	Coastal	0	12.08829	75.17603	India	Kavayi
203	mgm4667708.3	Coastal	0	9.996566	76.24719	India	Valpadananam
204	mgm4667773.3	Coastal	0	12.70833	74.90075	India	Bangramanjeshwar
205	mgm4667861.3	Coastal	0	12.5942	74.94662	India	Kumbla
206	mgm4671368.3	Coastal	0	9.895994	76.32609	India	Panangod
207	mgm4671369.3	Coastal	0	9.90919	76.30629	India	Madakal
208	mgm4671370.3	Coastal	0	12.10507	75.2058	India	Pyannur
209	mgm4671371.3	Coastal	0	9.999414	76.25371	India	Vallarpadam

Table S5: Sampling locations (n=410) across the Arabian seacoast (India) from seven biosystems.

State	Location	Latitude Longitude	Biosystem
Kerala	Manjeshwar beach	12°44'00.8"N 74°52'30.4"E	Seashore
Kerala	Cherangai beach,	12°30'57.0"N 74°58'05.3"E	Seashore
Kerala	Chembirika beach	12°26'44.0"N 75°00'03.7"E	Seashore
Kerala	Bekal fort	12°23'27.3"N 75°02'05.2"E	Seashore
Kerala	Kannuveedu kadapuram	12°06'42.6"N 75°09'20.0"E	Seashore
Kerala	Paddnakadappuram	12°09'45.9"N 75°08'06.9"E	Seashore
Kerala	Thaikadappuram, kerala	12°14'26.6"N 75°06'20.8"E	Seashore
Kerala	Valiyaparambu	12°08'20.9"N 75°08'51.7"E	River
Kerala	Thejaswani river	12°14'05.2"N 75°08'43.6"E	River
Kerala	Karyangod river	12°15'59.8"N 75°07'16.3"E	River
Kerala	Kuniyan river	12°06'57.4"N 75°11'25.0"E	River
Kerala	Valiyaparambu	12°07'31.8"N 75°09'32.7"E	Estuary
Kerala	Azhi pozhi	12°28'23.5"N 74°59'04.8"E	Estuary
Kerala	Bambrana-arikkadi	12°36'20.7"N 74°56'08.8"E	Mangrove
Kerala	Koyippady village	12°35'35.6"N 74°56'26.6"E	Mangrove
Kerala	Shiriya	12°37'00.7"N 74°55'57.8"E	Mangrove
Kerala	Madakkal	12°06'35.3"N 75°10'04.5"E	Mangrove
Kerala	Ettikulam beach	12°00'39.5"N 75°12'36.3"E	Seashore
Kerala	Mattool beach	11°59'37.6"N 75°15'38.5"E	Seashore
Kerala	Payyabalam beach	11°52'18.8"N 75°21'03.0"E	Seashore
Kerala	Thottada beach	11.838401"N 75°24'12.2"E	Seashore
Kerala	Chalad beach	11°52'49.3"N 75°20'41.0"E	Seashore
Kerala	Chootad beach	12°01'16.2"N 75°13'53.1"E	Seashore
Kerala	Muzhappilangad	11°47'46.3"N 75°26'31.5"E	Seashore
Kerala	Dharmadam beach	11°46'37.2"N 75°27'16.7"E	Seashore
Kerala	Kannur beach	11°51'26.3"N 75°23'00.3"E	Seashore
Kerala	Thalassery beach	11°44'47.3"N 75°29'12.9"E	Seashore
Kerala	Azhikkal	11°56'46.7"N 75°17'41.9"E	Estuary
Kerala	Dharmadam	11°46'39.4"N 75°27'20.8"E	Estuary
Kerala	Payyanur	12°05'20.7"N 75°11'30.8"E	Mangrove
Kerala	Chemballikundu	12°02'58.9"N 75°15'10.9"E	Mangrove
Kerala	Valapattanam	11°55'51.4"N 75°21'02.7"E	Mangrove
Kerala	Dharmadam	11°46'34.1"N 75°27'17.8"E	Mangrove
Kerala	Thalassery	11°46'02.7"N 75°28'57.9"E	Mangrove
Kerala	Kavvayi	12°05'53.2"N 75°10'42.1"E	Mangrove
Kerala	Perumba	12°05'24.2"N 75°11'09.8"E	River
Kerala	Kavvayi river	12°05'36.5 N 75°11'07.0"E	River
Kerala	Valapattanam river	11°56'06.5"N 75°20'47.7"E	River
Kerala	Mahe beach	11°42'14.2"N 75°31'50.1"E	Seashore
Kerala	Payyoli	11°30'42.9"N 75°36'32.5"E	Seashore
Kerala	Kappad	11°22'54.9"N 75°43'12.5"E	Seashore
Kerala	Kozhikode	11°15'45.8"N 75°45'56.8"E	Seashore
Kerala	Beypore	11°09'51.3"N 75°48'08.2"E	Seashore
Kerala	Kuruvaupuzha	11°16'44.6"N 76°12'07.9"E	River
Kerala	Chaliyar	11°09'39.0"N 75°48'20.4"E	River
Kerala	Mahe river	11°42'21.5"N 75°32'02.6"E	River
Kerala	Vadapyranpuzha	11°16'55.3"N 75°58'47.0"E	River
Kerala	Akalapuzha	11°20'13.7"N 75°44'58.3"E	River
Kerala	Cherupuzha	11°15'21.0"N 75°55'36.9"E	River
Kerala	Korapuzha	11°21'12.5"N 75°44'29.0"E	River
Kerala	Iruvanjippuzha	11°16'06.8"N 75°58'44.4"E	River
Kerala	Kallai	11°14'16.3"N 75°47'15.5"E	Estuary
Kerala	Kadalundi	11°07'53.7"N 75°49'49.6"E	Mangrove
Kerala	Koyilandy	11°26'06.2"N 75°43'44.0"E	Mangrove
Kerala	Kottooly	11°16'29.3"N 75°47'40.3"E	Mangrove
Kerala	Vallikunnu beach	11°05'31.2"N 75°50'17.1"E	Seashore
Kerala	Parappangandi beach	11°03'28.0"N 75°50'52.1"E	Seashore
Kerala	Kettungal beach	11°01'15.6"N 75°51'23.6"E	Seashore
Kerala	Tanur beach	10°58'58.6"N 75°51'54.4"E	Seashore
Kerala	Unniyal beach	10°55'05.5"N 75°52'56.6"E	Seashore
Kerala	Vakkal beach	10°52'44.2"N 75°53'25.7"E	Seashore
Kerala	Azheekkal beach	10°53'09.4"N 75°53'20.0"E	Seashore
Kerala	Padinjurekkara beach	10°47'42.8"N 75°54'32.9"E	Seashore
Kerala	Ponnani beach	10°47'06.8"N 75°54'44.8"E	Seashore
Kerala	Kappirikkad beach	10°41'20.7"N 75°57'19.7"E	Seashore
Kerala	Kadalundi azhimukkam	11°07'23.8"N 75°49'32.7"E	Estuary
Kerala	Ottumparam	11°01'11.8"N 75°51'24.7"E	Estuary
Kerala	Padinjurekkara	10°47'14.0"N 75°54'34.4"E	Estuary
Kerala	Ponnani river	10°48'48.1"N 75°54'52.4"E	River
Kerala	Chaliyar	11°15'51.0"N 75°58'56.7"E	River
Kerala	Kadalundi	11°08'19.1"N 75°50'30.0"E	River
Kerala	Tirur	10°47'51.2"N 75°54'48.2"E	River
Kerala	Bharathapuzha	10°50'21.3"N 76°05'11.0"E	River
Kerala	Beeyam kayal	10°44'38.2"N 75°56'16.9"E	Lake
Kerala	Periyambalam beach	10°40'49.2"N 75°57'33.6"E	Seashore
Kerala	Mannalamkunnu beach	10°39'29.9"N 75°58'11.8"E	Seashore
Kerala	Vaikkam nagar beach	10°38'56.4"N 75°58'26.9"E	Seashore
Kerala	Chavakkad	10°34'19.5"N 76°00'26.8"E	Seashore
Kerala	Kadappuram	10°32'42.9"N 76°01'14.1"E	Seashore
Kerala	Chettuva	10°30'20.2"N 76°02'26.6"E	Seashore
Kerala	Valapad beach	10°23'46.9"N 76°05'28.6"E	Seashore
Kerala	Palapetty beach	10°21'41.2"N 76°06'20.8"E	Seashore
Kerala	Puthiya beach	10°13'28.7"N 76°08'55.0"E	Seashore
Kerala	Munakkal beach	10°10'49.9"N 76°09'44.8"E	Seashore
Kerala	Puthuponnani	10°44'50.1"N 75°56'06.9"E	River
Kerala	Karuvannur river	10°31'11.1"N 76°02'21.9"E	River
Kerala	Periyar river	10°11'02.1"N 76°10'20.7"E	River
Kerala	Cheetuva river	10°30'40.2"N 76°02'23.3"E	River
Kerala	Cheetuva lake	10°33'03.6"N 76°02'56.2"E	Lake
Kerala	Chettuva	10°30'34.4"N 76°02'10.7"E	Estuary
Kerala	Azheekkodu	10°10'38.6"N 76°09'43.2"E	Estuary
Kerala	Puthuponnani	10°43'58.3"N 75°56'06.4"E	Estuary
Kerala	Cherai beach	10°08'22.3"N 76°10'44.0"E	Seashore
Kerala	Puthuvype beach	10°00'38.9"N 76°12'56.8"E	Seashore
Kerala	Kuzhupilly beach	10°06'34.7"N 76°11'14.7"E	Seashore
Kerala	Fort kochi beach	9°57'49.2"N 76°14'15.3"E	Seashore
Kerala	Njarackal arattuvazhi	10°02'12.2"N 76°12'29.1"E	Seashore
Kerala	Mahatma gandhi beach	9°58'00.9"N 76°14'23.2"E	Seashore
Kerala	Puthenthode beach	9°52'10.4"N 76°15'46.7"E	Seashore
Kerala	Veeranpuzha beach	10°04'12.3"N 76°14'24.4"E	Seashore
Kerala	Aluva	10°06'46.3"N 76°21'37.6"E	River
Kerala	Kaniyampuzha	9°57'40.9"N 76°19'33.7"E	River
Kerala	Chambakkara river	9°57'12.9"N 76°18'53.8"E	River
Kerala	Mattachery	9°58'04.6"N 76°15'09.0"E	Estuary
Kerala	Munakkal	10°10'42.9"N 76°09'44.9"E	Estuary
Kerala	Vembanad lake	9°58'29.1"N 76°16'41.8"E	Lake
Kerala	Pithuvypin	9°59'51.9"N 76°13'38.3"E	Mangrove
Kerala	Mangalvanam bird sanctuary	9°59'16.3"N 76°16'22.5"E	Mangrove
Kerala	Kundannur	9°56'27.0"N 76°18'42.4"E	Mangrove
Kerala	Mararikulam beach	9°36'02.6"N 76°17'53.3"E	Seashore
Kerala	Marari beach	9°36'02.7"N 76°17'53.7"E	Seashore
Kerala	Alappuzha beach	9°29'37.2"N 76°19'00.9"E	Seashore
Kerala	Andhakaranazhi beach	9°44'54.8"N 76°17'02.7"E	Seashore
Kerala	Thottappally beach	9°18'35.9"N 76°22'57.4"E	Seashore
Kerala	Pathiramanal beach	9°31'41.3"N 76°18'36.8"E	Seashore
Kerala	Arthunkal beach	9°43'03.8"N 76°17'15.0"E	Seashore
Kerala	Marai beach	9°35'20.9"N 76°17'59.3"E	Seashore
Kerala	Azhikkal	11°56'28.5"N 75°17'52.0"E	Seashore
Kerala	Paravoor	9°26'56.8"N 76°19'44.2"E	Seashore
Kerala	Valanjavazhy	9°23'46.6"N 76°20'45.7"E	Seashore
Kerala	Kayamkulam	9°11'23.8"N 76°26'40.0"E	Lake
Kerala	Pulinkunnu river	9°26'59.0"N 76°26'27.7"E	River
Kerala	Pambayar river	9°22'49.8"N 76°46'40.4"E	River
Kerala	Alappuzha river	9°30'44.1"N 76°24'26.4"E	River
Kerala	Achankovil	9°19'01.7"N 76°27'03.9"E	River
Kerala	Kayamkulam	9°09'49.8"N 76°27'39.4"E	Lake
Kerala	Punnamada	9°30'15.7"N 76°21'29.1"E	Lake
Kerala	Vembanad lake	9°36'59.9"N 76°22'13.1"E	Lake
Kerala	Kandalloor	9°10'26.4"N 76°27'46.7"E	Mangrove
Kerala	Thottappally estuary	9°18'39.1"N 76°22'56.8"E	Estuary
Kerala	Kollam beach	8°52'34.3"N 76°35'17.2"E	Seashore
Kerala	Thirumullavaram beach	8°53'42.0"N 76°33'13.0"E	Seashore
Kerala	Thangassery beach	8°52'53.7"N 76°34'09.4"E	Seashore
Kerala	Eravipuram beach	8°51'22.1"N 76°36'50.1"E	Seashore
Kerala	Mayyanad beach	8°49'23.7"N 76°38'30.1"E	Seashore
Kerala	Secret beach	8°53'43.6"N 76°33'11.8"E	Seashore
Kerala	Eravipuram beach	8°51'21.7"N 76°36'50.2"E	Seashore
Kerala	Chavara beach	8°58'21.5"N 76°31'37.4"E	Seashore
Kerala	Paravoor	8°47'31.5"N 76°39'55.5"E	Seashore
Kerala	Azheekkal	9°07'58.0"N 76°27'45.9"E	Seashore
Kerala	Mundakkal	8°52'02.8"N 76°36'03.5"E	Seashore
Kerala	Thottap	9°05'23.8"N 76°29'05.5"E	Seashore
Kerala	Needukara	8°57'35.7"N 76°31'51.6"E	Seashore
Kerala	Ozhukkathoda beach	8°54'16.7"N 76°32'48.0"E	Seashore
Kerala	Varkala beach	8°44'08.7"N 76°42'11.8"E	Seashore
Kerala	Ithikkara river	8°51'07.1"N 76°41'17.0"E	River
Kerala	Kallada river	9°04'55.0"N 76°44'58.8"E	River
Kerala	Pallikkal river	9°02'14.1"N 76°32'19.4"E	River
Kerala	Kureepuzha	8°55'16.8"N 76°33'59.0"E	River
Kerala	Ashtamudi lake	8°57'58.8"N 76°35'46.4"E	Lake
Kerala	Kattaka kayal	8°55'32.7"N 76°32'37.4"E	Lake
Kerala	Paravur lake	8°49'60.0"N 76°39'27.2"E	Lake
Kerala	Sasthamcotta lake	9°02'05.0"N 76°37'29.3"E	Lake
Kerala	Adventure park asramam	8°53'43.2"N 76°35'08.8"E	Mangrove
Kerala	Munrothuruth islands	8°59'05.2"N 76°36'56.4"E	Mangrove
Kerala	Kumbalam	8°59'40.4"N 76°39'23.4"E	Mangrove
Kerala	Mayyanad	8°49'14"N 76°38'41"E	Mangrove
Kerala	Paravur estuary	8°48'44.3"N 76°38'54.5"E	Estuary
Kerala	Shanghumugham beach	8°28'42.8"N 76°54'42.6"E	Seashore
Kerala	Puthenthope	8°34'19.8"N 76°50'08.4"E	Seashore
Kerala	Veli beach	8°30'44.6"N 76°53'03.1"E	Seashore
Kerala	Kovalam beach	8°23'20.2"N 76°58'33.6"E	Seashore
Kerala	Vizhinjam	8°22'35.8"N 76°59'41.2"E	Seashore
Kerala	Chowara beach	8°21'22.4"N 77°00'41.3"E	Seashore
Kerala	Poovar beach	8°18'36.0"N 77°04'25.7"E	Seashore
Kerala	Nellikunnu beach	8°36'51.2"N 76°48'02.8"E	Seashore
Kerala	Varkala beach	8°44'08.8"N 76°42'11.5"E	Seashore
Kerala	Anjuthengu	8°39'46.1"N 76°45'46.7"E	Seashore
Kerala	Pallithura	8°32'42.4"N 76°51'25.9"E	Seashore
Kerala	Valiathura beach	8°27'51.2"N 76°55'28.3"E	Seashore
Kerala	Parvati puthan river	8°26'41.4"N 76°56'55.2"E	River
Kerala	Neyyar	8°19'06.7"N 77°04'33.4"E	River
Kerala	Karamana river	8°27'17.6"N 76°57'49.0"E	River
Kerala	Vamanapuram river	8°42'04.1"N 76°55'56.9"E	River
Kerala	Ithikkara river	8°51'15.5"N 76°41'31.9"E	River
Kerala	Veli lake	8°30'36.9"N 76°53'20.0"E	Lake
Kerala	Aakulam lake	8°31'15.3"N 76°54'01.1"E	Lake
Kerala	Vellayani lake	8°26'02.3"N 76°59'41.2"E	Lake
Kerala	Vavvamoola lake	8°24'34.1"N 76°59'36.4"E	Lake
Kerala	Poovar	8°18'37.7"N 77°05'11.2"E	Mangrove
Kerala	Poovar estuary	8°18'32.6"N 77°04'36.4"E	Estuary
Lakshadweep	Agatti island	10°51'31.6"N 72°11'36.5"E	Island
Lakshadweep	Agatti island	10°51'31.6"N 72°11'36.5"E	Island
Lakshadweep	Agatti island	10°51'23.0"N 72°11'43.3"E	Island
Lakshadweep	Chetlat island	11°41'21.2"N 72°42'51.2"E	Island
Lakshadweep	Kiltān island	11°28'59.3"N 73°00'13.3"E	Island
Lakshadweep	Kiltān island	11°28'44.8"N 73°00'16.5"E	Island
Lakshadweep	Kadmat island	11°12'50.0"N 72°46'33.8"E	Island
Lakshadweep	Amini island 1	11°06'48.8"N 72°43'14.9"E	Island
Lakshadweep	Androth island	11°06'49.3"N 72°43'05.5"E	Island
Lakshadweep	Kavaratti island	10°33'17.3"N 72°38'00.9"E	Island
Lakshadweep	Kavaratti island	10°33'29.5"N 72°38'33.2"E	Island
Lakshadweep	Kalpeni island	10°03'57.0"N 73°38'17.0"E	Island
Lakshadweep	Minicoy island	8°16'26.4"N 73°02'23.9"E	Island
Lakshadweep	Minicoy island	8°16'36.7"N 73°02'51.0"E	Island
Lakshadweep	Bangaram island	10°56'31.6"N 72°17'19.6"E	Island
Goa	Anjuna beach, goa	15°34'24.4"N 73°44'27.1"E	Seshore
Goa	Anjuna beach, goa	15°34'24.9"N 73°44'26.8"E	Seshore
Goa	Baga beach	15°33'18.9"N 73°45'04.9"E	Seshore
Goa	Baga beach	15°33'18.1"N 73°45'04.9"E	Seshore
Goa	Dona paula beach	15°27'17.4"N 73°48'16.0"E	Seshore
Goa	Dona paula beach	15°27'17.2"N 73°48'15.1"E	Seshore
Goa	Covla beach	15°16'50.0"N 73°54'42.3"E	Seshore
Goa	Covla beach	15°16'48.3"N 73°54'42.7"E	Seshore
Goa	Covla beach	15°16'48.3"N 73°54'42.7"E	Seshore
Goa	Covla beach	15°16'33.9"N 73°54'47.6"E	Seshore
Goa	Morber beach	15°09'26.6"N 73°56'41.7"E	Seshore
Goa	Morber beach	15°09'21.5"N 73°56'41.2"E	Seshore
Goa	Paloelum beach	15°00'35.9"N 74°01'23.7"E	Seshore
Goa	Paloelum beach	15°00'38.4"N 74°01'19.7"E	Seshore
Goa	Pollem beach	14°54'30.0"N 74°04'54.7"E	Seshore
Goa	Pollem beach	14.908269, 74.081926	Seshore
Goa	Pollem beach	14.908090, 74.082100	Seshore
Goa	Varca beach, fatrade	15°13'16.4"N 73°55'44.0"E	Seshore
Goa	Varca beach, fatrade	15°13'13.1"N 73°55'43.9"E	Seshore
Goa	Carmona beach, fatrade	15°12'03.7"N 73°56'01.9"E	Seshore
Goa	Chapora river	15°37'23.7"N 73°44'25.5"E	River
Goa	Mandovi river	15°30'24.5"N 73°54'24.9"E	River
Goa	Mandovi river	15°30'17.1"N 73°53'24.5"E	River
Goa	Mapusa river	15°31'15.5"N 73°50'58.9"E	River
Goa	Mapusa river	15°31'02.2"N 73°50'51.9"E	River
Goa	Sal river	15°10'56.9"N 73°57'48.5"E	River
Goa	Terekhol river	15°49'12.0"N 73°51'15.2"E	River
Goa	Zuari river	15°24'15.2"N 73°55'24.0"E	River
Goa	Zuari river	15°24'12.1"N 73°56'01.2"E	River
Goa	Mandovi	15°30'19.2"N 73°53'20.3"E	Mangrove
Goa	Zuari rivers	15°24'09.0"N 73°55'48.3"E	Mangrove
Karnataka	Malpe beach	13°21'24.0"N 74°41'53.4"E	Seashore
Karnataka	Tagore beach	14°48'38.7"N 74°07'31.8"E	Seashore
Karnataka	Devbagh beach	14°50'53.1"N 74°06'42.3"E	Seashore
Karnataka	Eco beach	14°15'38.5"N 74°25'58.1"E	Seashore
Karnataka	Gokarna beach	14°32'36.3"N 74°18'51.3"E	Seashore
Karnataka	Karwar beach	14°49'53.8"N 74°07'28.7"E	Seashore
Karnataka	Kaup beach	13°13'23.8"N 74°44'26.2"E	Seashore
Karnataka	Kudle beach, gokarna	14°31'55.3"N 74°18'44.6"E	Seashore
Karnataka	Kumta	14°25'08.9"N 74°23'24.0"E	Seashore
Karnataka	Kundapura beach	13°38'12.2"N 74°40'03.2"E	Seashore
Karnataka	Malpe beach	13°21'24.3"N 74°41'51.6"E	Seashore
Karnataka	Maravanthe beach	13°42'17.3"N 74°38'33.0"E	Seashore
Karnataka	Maravanthe beach	13°42'35.6"N 74°38'30.1"E	Seashore
Karnataka	Murudeshwar beach	14°05'41.8"N 74°29'03.0"E	Seashore
Karnataka	Murudeshwar beach	14°05'43.6"N 74°29'08.6"E	Seashore
Karnataka	Om beach	14°31'07.4"N 74°17'55.2"E	Seashore
Karnataka	Om beach, gokarna	14°32'21.8"N 74°18'41.7"E	Seashore
Karnataka	Panambur beach	12°56'10.6"N 74°48'16.4"E	Seashore
Karnataka	Panambur beach	14°31'09.3"N 74°19'22.7"E	Seashore
Karnataka	Tannirbhavi beach	12°53'57.0"N 74°48'43.3"E	Seashore
Karnataka	Surathkal beach	13°00'34.7"N 74°47'21.8"E	Seashore
Karnataka	Ullal beach	12°48'27.3"N 74°50'33.7"E	Seashore
Karnataka	Kodi beach	13°36'00.6"N 74°40'22.7"E	Seashore
Karnataka	Kali river	14°52'23.0"N 74°11'47.5"E	River
Karnataka	River gangavali	14°36'24.9"N 74°20'03.4"E	River
Karnataka	Aghanashini	14°31'18.5"N 74°22'35.1"E	River
Karnataka	Badagani	14°18'43.5"N 74°25'17.7"E	River
Karnataka	Sharavati river	14°15'36.0"N 74°27'37.2"E	River
Karnataka	Venkatapur river	14°01'40.7"N 74°30'28.5"E	River
Karnataka	Pallinadi river	14°01'43.2"N 74°31'06.3"E	River
Karnataka	Chowtani river	13°58'03.7"N 74°32'10.1"E	River
Karnataka	Baindur river	13°51'20.7"N 74°36'49.7"E	River
Karnataka	Yadamavu river	13°48'13.0"N 74°37'29.0"E	River
Karnataka	Netravati river	12°50'34.0"N 74°50'37.2"E	River
Karnataka	Honnavar	14°16'38.7"N 74°26'27.6"E	Estuary
Karnataka	Gangolii estuary	13°37'54.3"N 74°40'00.2"E	Estuary
Karnataka	Nakhuda	14°01'35.0"N 74°30'05.9"E	Estuary
Karnataka	Adam kudru	12°50'04.7"N 74°51'29.5"E	Mangrove
Karnataka	Udupi	13°05'59.5"N 74°47'42.3"E	Mangrove
Karnataka	Mulk	13°03'58.3"N 74°46'54.9"E	Mangrove
Maharashtra	Aare ware beach	17°04'38.0"N 73°17'22.9"E	Seshore
Maharashtra	Alibaug	18°38'26.6"N 72°52'12.4"E	Seshore
Maharashtra	Anjarle beach	18°38'28.1"N 72°52'10.6"E	Seshore
Maharashtra	Chowpatty beach	18°57'17.2"N 72°48'33.3"E	Seshore
Maharashtra	Dana pani beach	19°10'10.5"N 72°47'18.1"E	Seshore
Maharashtra	Devgad beach	16°22'37.5"N 73°22'14.4"E	Seshore
Maharashtra	Diveagar beach	18°11'32.4"N 72°58'41.2"E	Seshore
Maharashtra	Ganeshgule beach	16°52'00.5"N 73°17'37.4"E	Seshore
Maharashtra	Ganpatipule	17°08'54.0"N 73°15'51.6"E	Seshore
Maharashtra	Guhagar beach	17°29'20.3"N 73°11'04.9"E	Seshore
Maharashtra	Harihareshwar beach	18°00'13.1"N 73°01'01.8"E	Seshore
Maharashtra	Hedvi ganesh	17°28'09.6"N 73°11'36.0"E	Seshore
Maharashtra	Juhu beach	19°05'50.9"N 72°49'35.2"E	Seshore
Maharashtra	Kashid beach	18°26'48.1"N 72°54'09.2"E	Seshore
Maharashtra	Kelshi beach	17°55'10.4"N 73°03'14.5"E	Seshore
Maharashtra	Kihim beach	18°43'22.7"N 72°51'49.7"E	Seshore
Maharashtra	Kondivli beach	18°06'06.5"N 72°59'16.1"E	Seshore
Maharashtra	Korlai fort	18°32'11.2"N 72°54'33.0"E	Seshore
Maharashtra	Mandwa beach	18°48'11.4"N 72°52'41.7"E	Seshore
Maharashtra	Murud beach	18°19'46.6"N 72°57'15.6"E	Seshore
Maharashtra	Ratnagiri	16°59'23.7"N 73°17'09.8"E	Seshore
Maharashtra	Revdanda fort	18°32'48.6"N 72°55'28.5"E	Seshore
Maharashtra	Shrivardhan beach	18°02'30.2"N 73°00'39.4"E	Seshore
Maharashtra	Velas beach	17°57'42.5"N 73°01'43.5"E	Seshore
Maharashtra	Velneshwar	17°22'51.4"N 73°12'31.1"E	Seshore
Maharashtra	Bhudal beach	17°24'25.1"N 73°10'27.7"E	Seshore
Maharashtra	Wayari bhutnath beach	16°02'34.1"N 73°28'40.1"E	Seshore
Maharashtra	Wayari beach	16°02'19.2"N 73°28'53.3"E	Seshore
Maharashtra	Tarkarli beach	16°00'49.8"N 73°29'23.4"E	Seshore
Maharashtra	Mumbai fort	18°55'55.1"N 72°50'23.9"E	Seshore
Maharashtra	Kundalika river	18°26'23.1"N 73°07'38.5"E	River
Maharashtra	Savitri river	17°59'13.2"N 73°07'21.8"E	River
Maharashtra	Vashishti	17°59'13.2"N 73°07'21.8"E	River
Maharashtra	Mithi river	19°03'26.4"N 72°52'04.3"E	River
Maharashtra	Vaitama river	19°33'54.2"N 72°52'41.9"E	River
Maharashtra	Banganga-1	19°48'11.8"N 72°41'44.6"E	River
Maharashtra	Vihar lake	19°09'12.1"N 72°54'02.5"E	Lake
Maharashtra	Tansa	19°34'16.3"N 73°16'32.6"E	Lake
Maharashtra	Powai lake	19°07'28.3"N 72°53'51.5"E	Lake
Maharashtra	Virar	19°29'33.7"N 72°46'59.0"E	Estuary
Maharashtra	Ratnagiri	17°34'37.5"N 73°10'06.0"E	Estuary
Maharashtra	Vijaydurg	16°33'37.7"N 73°20'04.2"E	Estuary
Maharashtra	Mulund	19°09'07.0"N 72°57'34.0"E	Saltpan
Maharashtra	Mira bhayandar	19°18'07.4"N 72°52'42.3"E	Saltpan
Maharashtra	Aagri	19°17'32.1"N 72°48'34.7"E	Saltpan
Maharashtra	Kandarpada	19°15'33.7"N 72°51'01.0"E	Mangrove
Maharashtra	Ganpat patil nagar	19°15'29.0"N 72°50'59.4"E	Mangrove
Maharashtra	Ganpat patil nagar	19°15'19.7"N 72°50'21.5"E	Mangrove
Maharashtra	Uttan	19°15'00.4"N 72°49'45.4"E	Mangrove
Maharashtra	Manori creek	19°12'50.5"N 72°48'36.5"E	Mangrove
Maharashtra	Manori	19°13'13.9"N 72°48'07.2"E	Mangrove
Maharashtra	Uttan	19°18'15.8"N 72°48'01.5"E	Mangrove
Maharashtra	Andheri west	19°09'08.7"N 72°48'59.8"E	Mangrove
Maharashtra	Andheri west	19°09'01.7"N 72°49'14.0"E	Mangrove
Maharashtra	Paili hill	19°04'00.4"N 72°49'17.1"E	Mangrove
Maharashtra	Mithi river side	19°03'05.7"N 72°50'45.2"E	Mangrove
Maharashtra	Mithi river side	19°03'04.1"N 72°50'49.5"E	Mangrove
Maharashtra	Paili hill	19°04'05.0"N 72°49'18.2"E	Mangrove
Maharashtra	Andheri west	19°09'06.7"N 72°49'16.7"E	Mangrove
Maharashtra	Uttan	19°18'17.2"N 72°48'01.9"E	Mangrove
Maharashtra	Manori	19°12'43.1"N 72°47'50.4"E	Mangrove
Maharashtra	Manori creek	19°12'44.3"N 72°48'30.9"E	Mangrove
Maharashtra	Kandarpada	19°15'26.9"N 72°50'58.7"E	Mangrove
Gujarat	Chakratirth beach	20°42'14.4"N 70°58'34.0"E	Seashore
Gujarat	Dandi beach, surat	20°53'11.8"N 72°47'50.0"E	Seashore
Gujarat	Devka beach	20°26'58.0"N 72°50'09.7"E	Seashore
Gujarat	Dumas beach	21°05'28.3"N 72°42'15.4"E	Seashore
Gujarat	Dwarka beach	22°14'24.2"N 68°57'26.3"E	Seashore
Gujarat	Ghogla beach	20°44'25.9"N 71°00'34.4"E	Seashore
Gujarat	Gomtimata beach	20°42'32.8"N 70°52'26.1"E	Seashore
Gujarat	Gopnath beach	21°12'00.6"N 72°06'31.7"E	Seashore
Gujarat	Jallandhar beach	20°42'32.8"N 70°59'07.4"E	Seashore
Gujarat	Mandvi beach	22°49'50.1"N 69°19'33.3"E	Seashore
Gujarat	Nagoa beach	20°42'44.2"N 70°55'58.2"E	Seashore
Gujarat	Nargol beach	20°13'32.6"N 72°44'42.5"E	Seashore
Gujarat	Okha beach	22°05'05.3"N 69°06'02.5"E	Seashore
Gujarat	Porbandar	21°37'40.2"N 69°36'45.3"E	Seashore
Gujarat	Positra beach	22°23'57.0"N 69°10'00.8"E	Seashore
Gujarat	Sarkeshwar	20°50'03.9"N 71°17'39.8"E	Seashore
Gujarat	Somnath beach	20°53'08.7"N 70°24'18.7"E	Seashore
Gujarat	Suvali beach, surat	21°09'49.9"N 72°37'06.6"E	Seashore
Gujarat	Tithal beach, surat	20°35'52.4"N 72°53'41.6"E	Seashore
Gujarat	Ubharat beach	21°01'21.7"N 72°43'41.7"E	Seashore
Gujarat	Mangrol	21°06'16.6"N 70°06'21.9"E	Seashore
Gujarat	Vanakbara beach	20°42'33.5"N 70°53'14.8"E	Seashore
Gujarat	Naliya mandvi	20°44'19.8"N 71°01'28.1"E	Seashore
Gujarat	Sarkeshwar beach	20°50'04.4"N 71°17'39.5"E	Seashore
Gujarat	Dandi beach	20°53'12.1"N 72°47'49.9"E	Seashore
Gujarat	Diu	20°42'16.8"N 70°58'32.1"E	Seashore
Gujarat	Dandi beach, surat	20°53'08.7"N 72°47'49.6"E	Seashore
Gujarat	Bhimpore	20°26'56.4"N 72°50'04.6"E	Seashore
Gujarat	Devka beach	20°26'36.0"N 72°49'54.0"E	Seashore
Gujarat	Dwarka beach	22°14'29.6"N 68°57'23.1"E	Seashore
Gujarat	Ghogla beach	20°44'19.5"N 71°00'36.8"E	Seashore
Gujarat	Vanakbara beach	20°42'37.2"N 70°52'25.7"E	Seashore
Gujarat	Vanakbara beach	20°42'32.3"N 70°52'59.5"E	Seashore
Gujarat	Mangrol	21°06'19.5"N 70°06'17.7"E	Seashore
Gujarat	Chakratirth beach	20°42'15.9"N 70°58'32.0"E	Seashore
Gujarat	Nagoa beach	20°42'37.4"N 70°56'15.4"E	Seashore
Gujarat	Nargol beach	20°13'17.5"N 72°44'44.8"E	Seashore
Gujarat	Okha beach	22°04'24.6"N 69°06'35.3"E	Seashore
Gujarat	Porbandar	21°37'39.2"N 69°36'46.2"E	Seashore
Gujarat	Devbhoomi dwarka	22°24'29.6"N 69°11'33.9"E	Seashore
Gujarat	Amreli	20°50'01.5"N 71°17'35.5"E	Seashore
Gujarat	Lakhota lake	22°28'02.8"N 70°03'56.8"E	Lake
Gujarat	Kankaria lake	23°00'29.7"N 72°36'11.0"E	Lake
Gujarat	Gopi talav	21°11'21.5"N 72°49'48.1"E	Lake
Gujarat	Nal sarovar	22°48'35.2"N 72°03'02.3"E	Lake
Gujarat	Narayan sarovar	23°40'28.4"N 68°32'26.3"E	Lake
Gujarat	Damodar kund	21°31'32.4"N 70°29'11.5"E	Lake
Gujarat	Sursagar lake	22°18'03.6"N 73°12'19.4"E	Lake
Gujarat	Thol lake	23°08'54.4"N 72°24'20.6"E	Lake
Gujarat	Chandola lake	22°59'27.9"N 72°35'06.9"E	Lake
Gujarat	Lakhota lake	22°27'48.8"N 70°03'50.1"E	Lake
Gujarat	Bharuch	22°14'19.6"N 72°52'06.2"E	Estuary
Gujarat	Dahej	21°52'10.2"N 72°32'40.1"E	Estuary
Gujarat	Aliabet	21°37'17.3"N 72°39'06.5"E	Estuary
Gujarat	Amreli	20°57'55.4"N 71°31'04.7"E	Mangrove
Gujarat	Amreli	20°57'44.4"N 71°30'49.7"E	Mangrove
Gujarat	Limla	21°08'59.0"N 72°42'17.5"E	Mangrove
Gujarat	Hazira creek	21°09'12.9"N 72°40'08.8"E	Mangrove
Gujarat	Taria	21°40'49.3"N 72°53'06.7"E	Mangrove
Gujarat	Taria	21°40'31.3"N 72°53'14.0"E	Mangrove
Gujarat	Narmadariver	21°40'53.8"N 72°53'00.4"E	River
Gujarat	Narmadariver	21°39'59.2"N 72°55'03.6"E	River
Gujarat	Narmadariver	21°38'23.0"N 72°42'23.5"E	River
Gujarat	Tapi river	21°08'24.1"N 72°44'11.9"E	River
Gujarat	Tapi river	21°08'39.0"N 72°44'41.2"E	River
Gujarat	Tapi river	21°09'16.3"N 72°45'34.2"E	River
Gujarat	Purna river	20°57'15.7"N 72°50'03.9"E	River
Gujarat	Purna river	20°57'09.4"N 72°50'39.3"E	River
Gujarat	Purna river	20°56'48.1"N 72°51'04.1"E	River
Gujarat	Ambika river	20°44'32.4"N 72°54'05.4"E	River
Gujarat	Ambika river	20°44'48.2"N 72°54'51.4"E	River
Gujarat	Ambika river	20°45'08.7"N 72°55'25.9"E	River
Gujarat	Kharera river	20°44'25.8"N 72°58'40.3"E	River
Gujarat	Kharera river	20°44'31.6"N 72°58'31.0"E	River
Gujarat	Auranga river	20°35'48.9"N 72°58'04.0"E	River
Gujarat	Wanki river	20°37'53.3"N 72°53'29.2"E	River
Gujarat	Par river	20°31'37.7"N 72°55'06.5"E	River
Gujarat	Kolak river	20°27'47.3"N 72°52'04.3"E	River
Gujarat	Daman ganga river	20°24'45.6"N 72°50'18.8"E	River
Gujarat	Daman ganga	20°24'38.6"N 72°50'13.4"E	River
Tamil Nadu	Kanyakumari	8°05'11.8"N 77°33'16.0"E	Seashore

Script: The R script for checking the presence of required gene(s)

```r
library("rjson")
library("httr")
library("htmltools")
library("svMisc")
library("RMySQL")
library(DBI)
library(pool)
library(tidyverse)

#get the total number of WGS
port_no <- 3306
pool <- dbPool(drv = RMySQL::MySQL(), dbname = "eztaxon_db", host = "localhost", username = "root", password = "", port = port_no, unix.sock = "/var/run/mysqld/mysqld.sock")
total_wgs_bac <- 22615 #dbGetQuery(pool, "select count(distinct puid) from eztaxon_wgs_bulk")
total_wgs_arc <- 925 #dbGetQuery(pool, "select count(distinct puid) from eztaxon_wgs_bulk_arc")

#open the raw file with cruF genes (example)
path <- dirname(rstudioapi::getSourceEditorContext()$path)
df = read.delim(paste0(path, "/Selected_genes/Bac_CruF.csv"), header = FALSE, stringsAsFactors = FALSE, quote = "", sep = ",")
colnames(df)=c("id","puid","taxon_name","ncbi_name","name","feature_name","feature_key","feature_index","gene","ref_feature_name","contig_index","contig_acc","begin","end","iscomp","title","color","pseudo_chromosome","location","product","note",[illegible]
unction_qaz","seed_figfam_id","seed_function","kegg_id","kegg_function","eggnog_id","eggnog_function","go_id","ec_number","db_xref","dna","translation","added_on" )

#extract alll unique species
df_unique <- df %>%  distinct(ncbi_name, .keep_all = TRUE)

#filter only halobacteria
halobacteria_puid <- dbGetQuery(pool, "select puid from taxonomy_arc WHERE Calssification LIKE '%;halobacteria;%'")

#extract halobacteria species
df_selected <- df_unique[df_unique$puid %in% c(halobacteria_puid)$puid,]

stat_total_gene <- nrow(df)
stat_unique_strains <- nrow(df_unique)
stat_unique_halobacteria_class_strains <- nrow(df_selected)

write.table( paste0("Bac_CruF", "\nTotal Gene: ", stat_total_gene,  "\nUnique Strains: ", stat_unique_strains),"clipboard-16384",sep="\t", row.names = FALSE)

# Bac_CruF
# Total Gene: 397
# Unique Strains: 382
```
